# Protein Targets of Acetaminophen Covalent Binding in Rat and Mouse Liver Studied by LC-MS/MS

**DOI:** 10.3389/fchem.2021.736788

**Published:** 2021-08-20

**Authors:** Timon Geib, Ghazaleh Moghaddam, Aimee Supinski, Makan Golizeh, Lekha Sleno

**Affiliations:** Chemistry Department, Université du Québec à Montréal, Montréal, QC, Canada

**Keywords:** acetaminophen, rodent model, reactive metabolites, liver proteins, LC-MS/MS, LC-MRM, protein modification, NAPQI, N-acetyl-p-benzoquinone, mouse, rat, covalent binding

## Abstract

Acetaminophen (APAP) is a mild analgesic and antipyretic used commonly worldwide. Although considered a safe and effective over-the-counter medication, it is also the leading cause of drug-induced acute liver failure. Its hepatotoxicity has been linked to the covalent binding of its reactive metabolite, *N*-acetyl *p*-benzoquinone imine (NAPQI), to proteins. The aim of this study was to identify APAP-protein targets in both rat and mouse liver, and to compare the results from both species, using bottom-up proteomics with data-dependent high resolution mass spectrometry and targeted multiple reaction monitoring (MRM) experiments. Livers from rats and mice, treated with APAP, were homogenized and digested by trypsin. Digests were then fractionated by mixed-mode solid-phase extraction prior to liquid chromatography-tandem mass spectrometry (LC-MS/MS). Targeted LC-MRM assays were optimized based on high-resolution MS/MS data from information-dependent acquisition (IDA) using control liver homogenates treated with a custom alkylating reagent yielding an isomeric modification to APAP on cysteine residues, to build a modified peptide database. A list of putative *in vivo* targets of APAP were screened from data-dependent high-resolution MS/MS analyses of liver digests, previous *in vitro* studies, as well as selected proteins from the target protein database (TPDB), an online resource compiling previous reports of APAP targets. Multiple protein targets in each species were found, while confirming modification sites. Several proteins were modified in both species, including ATP-citrate synthase, betaine-homocysteine *S*-methyltransferase 1, cytochrome P450 2C6/29, mitochondrial glutamine amidotransferase-like protein/ES1 protein homolog, glutamine synthetase, microsomal glutathione *S*-transferase 1, mitochondrial-processing peptidase, methanethiol oxidase, protein/nucleic acid deglycase DJ-1, triosephosphate isomerase and thioredoxin. The targeted method afforded better reproducibility for analysing these low-abundant modified peptides in highly complex samples compared to traditional data-dependent experiments.

## Introduction

Drugs are generally metabolized by the liver into biologically inactive forms and eliminated from the body through bile and urine. However during these processes, they can also be bioactivated by hepatic enzymes into reactive electrophilic intermediates and subsequently react with nucleophilic sites of proteins to form covalent adducts (Xu et al., 2005; Park et al., 2011). Reactive metabolites can in turn cause oxidative stress, depleting intracellular glutathione, and affect energy metabolism by binding to mitochondrial protein complexes (Pessayre, 1995; Srivastava et al., 2010).

Acetaminophen (*N*-acetyl *p*-aminophenol, APAP) is one of the most commonly used analgesic and antipyretic, and is known to be the number one cause of acute liver failure in North America (Bateman, 2016). APAP is deemed safe as long as recommended doses are not exceeded, but very high doses can cause severe hepatotoxicity, liver injury and even death in both children and adults (Tittarelli et al., 2017). APAP metabolism primarily occurs in the liver, where major biotransformations include sulfate and glucuronide conjugation forming readily excretable phase II metabolites. A smaller proportion, between 10 and 15%, is converted to the reactive electrophile *N*-acetyl *p*-benzoquinone imine (NAPQI), by multiple cytochrome P450 enzymes (Laine et al., 2009). NAPQI can generally be detoxified *via* glutathione conjugation and further excreted *via* the mercapturic acid pathway. However, excess NAPQI can bind to nucleophilic sulfhydryl groups in cysteines of cellular proteins, leading to possible protein dysfunction due to conformational changes, potentially leading to acute liver failure (James et al., 2003; Graham and Scott, 2005). Mitochondrial protein adducts have been shown to directly correlate to the initiation of hepatotoxicity through mitochondrial dysfunction and DNA fragmentation (Tirmenstein and Nelson, 1989; McGill et al., 2012; McGill et al., 2014; Xie et al., 2015; Ramachandran and Jaeschke, 2020). Identifying and monitoring modified proteins from *in vivo* samples could help better understand the metabolic processes involved in APAP-related hepatotoxicity and potentially lead to the detection of important biomarkers (Cohen et al., 1997; Bissell et al., 2001).

In this study, liquid chromatography coupled to tandem mass spectrometry (LC-MS/MS) was employed to identify *in vivo* protein targets of APAP, *via* the formation of NAPQI, in mouse and rat. Liver homogenates were digested by trypsin and fractionated by mixed-mode solid-phase extraction (SPE) prior to LC-MS/MS analysis. A scheduled multiple reaction monitoring (MRM) was developed based on data-dependent LC-MS/MS results as well as previous reports of APAP-modified proteins, while using a custom alkylation reagent to prepare isomerically-modified peptides from liver homogenates. This comparative analysis in rat and mouse could potentially provide novel mechanistic insight into APAP-related hepatotoxicity.

## Materials and Methods

### Chemicals and Materials

Urea was purchased from BioRad (Mississauga, ON, Canada). APAP, trypsin (TPCK-treated, from bovine pancreas), sodium *n*-dodecyl sulfate (SDS), dithiothreitol (DTT), iodoacetamide (IAM), thiourea, ammonium bicarbonate, ammonium acetate, ammonium hydroxide, formic acid, acetic acid, acetonitrile (ACN), methanol (MeOH) and all other reagents were from Sigma-Aldrich (St. Louis, MO, United States). Labeling agent *N*-(4-hydroxyphenyl)-2-iodoacetamide (HP-IAM) was synthesized in-house as previously described (LeBlanc et al., 2014). Ultra-pure water was produced using a Millipore Synergy UV system (Billerica, MA, United States).

### *In Vivo* Experiments

Four Sprague-Dawley male rats (450−550 g) were dosed with 600 mg/kg APAP (IP; solubilized in 60% PEG 200) or two animals were treated with vehicle for control samples. Rat livers were collected after 24 h post dosing. Male C57BL/6 mice (27−35 g) were treated (IP, in saline) with 150 mg/kg (2 and 6 h) and 300 mg/kg (2 and 6 h), as well as with vehicle for control samples. Two mice were treated at each dose and timepoint. All experiments were performed at *INRS Centre National de Biologie Expérimentale* (Laval, QC, Canada). The protocol was approved by the Ethics Committee of the *INRS Centre National de Biologie Expérimentale* under the ethical practices of the Canadian Council on Animal Care (project UQLK.14.02).

### Sample Preparation

Frozen liver samples, stored at −80°C, were homogenized in 100 mM ammonium bicarbonate (ABC buffer, pH 8–8.5) at 5 ml/g tissue weight using a hand-held homogenizer (Tissuemiser; Thermo Fisher Scientific, Waltham, MA, United States), and 100 μl aliquots were combined with 25 μl of 0.1% SDS, and heated for 5 min at 95°C. After cooling samples, 50 μl of a solution of 7 M urea and 2 M thiourea in water was added, and a probe sonicator was used ((XL-2000; Qsonica, Newtown, CT, United States) for three cycles of 10 s, followed by another 15 min in an ultrasonic bath (Branson 2510; Branson Ultrasonics, Brookfield, CT, United States). The resulting protein extract was then diluted with 500 μl of 100 mM ABC buffer. Reductive alkylation was performed by adding DTT (30 μl, 100 mM; 37°C for 20 min), followed by IAM (45 μl, 100 mM; 37°C for 30 min in the dark). Similarly, control samples, from vehicle-treated animals, were alkylated using HP-IAM (by simply substituting the IAM mentioned above). Samples were then digested by trypsin (30 μl, 1 mg/ml; 37°C for 18 h), followed by the addition of 300 μl of 2% formic acid prior to fractionation by solid phase extraction (SPE) on OASIS MCX cartridges (1 cc, 30 mg; Waters, Milford, MA, United States). Loaded cartridges were washed in three steps with 2% formic acid in water, 100% MeOH and 50% MeOH (1 ml each). Eight fractions were collected by eluting with 15, 20, 25, 35, 50, and 200 mM ammonium acetate in 50% MeOH, then 0.1 and 3% ammonium hydroxide in 50% MeOH (1 ml each). Fractions were dried under vacuum and stored at −30°C until analysis.

### LC-MS/MS Analysis

SPE fractions were reconstituted in 100 µl 10% acetonitrile and injected (20 μl) onto an Aeris PEPTIDE XB**-**C18 column (100 × 2.1 mm, 1.7 μm) (Phenomenex, Torrance, CA, United States) using a Nexera UHPLC system (Shimadzu, Columbia, MD, United States) with water (A) and ACN (B), both containing 0.1% formic acid, at a flow rate of 300 μl/min (at 40°C). The elution gradient started at 5% B for 2.5 min, and was linearly increased to 30% B in 47.5 min, to 50% B in 5 min, then to 90% B in 1 min, held for another 4 min at 90% B.

Data-dependent experiments were performed to collect MS and MS/MS spectra on a high-resolution quadrupole-time-of-flight TripleTOF 5600 mass spectrometer (Sciex, Concord, ON, Canada) equipped with a DuoSpray ion source in positive electrospray mode set at 5 kV source voltage, 500°C source temperature and 50 psi GS1/GS2 gas flows, with a declustering potential of 80 V. The instrument performed a survey TOF-MS acquisition from *m/z* 140–1250 (250 ms accumulation time), followed by MS/MS on the 15 most intense precursor ions with a minimum intensity of 250 cps from *m/z* 300–1250. Precursor ion were excluded for 20 s after two occurrences in data-dependent acquisition (DDA) mode with dynamic background subtraction. Each MS/MS acquisition (*m/z* 80–1500, high sensitivity mode) had an accumulation time of 50 ms and collision energy of 30 ± 10 V, with a total cycle time was 1.05 s. The MS instrument was calibrated at every four injections using an in-house calibration mix. Analyst TF software (1.7.1; Sciex) was used for data acquisition and raw data was visualized with PeakView 2.2 with MasterView 1.1 (Sciex). The mass spectrometry proteomics data from high-resolution data-dependent expriments have been deposited to the ProteomeXchange Consortium *via* the PRIDE (Perez-Riverol et al., 2019) partner repository with the dataset identifier PXD027674 and 10.6019/PXD027674.

Targeted assays were developed using scheduled LC-MRM on a Sciex QTRAP 5500 hybrid quadrupole-linear ion trap system with a TurboIonSpray ion source in positive mode, with identical UHPLC conditions as for data-dependent high resolution experiments. Source parameters were as follows: ionspray voltage 5 kV; temperature 550°C; GS1 and GS2 50 psi; and curtain gas 35 psi. Declustering, entrance and collision cell exit potentials were set at 80, 10 and 13 V, respectively. Collision-induced dissociation was performed at a collision energy of 30 V. Scheduled MRM time windows were set at 240 s, with a targeted scan time at 1.25 s. Minimum and maximum dwell times were 10 and 250 ms, respectively. MRM transitions monitored for SPE fractions two to eight can be found in Supplementary Tables S1, S2, for rat and mouse, respectively. Sciex Analyst software 1.7 was used for data acquisition.

### Data Processing

Raw data files from quadrupole-time-of-flight experiments were searched against the rat or mouse UniProtKB/Swiss-Prot protein database (release date: 07/18/2018, including common protein contaminants) using Sciex ProteinPilot 5.0. To detect APAP covalent adducts, a custom modification was added to the Paragon algorithm (Shilov et al., 2007) with a probability of 50% for APAP modification on cysteine residues (C_8_H_8_NO_2_ replacing H, *Δm* = +149.04766 u). The search was performed for +2 to +4 charge states at a MS tolerance of 0.05 u on precursor ions and 0.1 u on fragments. For protein identifications, a target-decoy approach was applied at a 1% false discovery rate (Aggarwal and Yadav, 2016). From the list of identified APAP-modified peptides, those with spectral confidence less than 95% were removed, as were those with modifications other than APAP and cysteine carbamidomethylation (CAM).

LC-MRM peaks were integrated and verified using MultiQuant 3.0.2 (Sciex) to ensure that APAP-modified peptides matched with *N*-(4-hydroxyphenyl)-2-carbamidomethylated (HP-CAM) peptides from control samples. Confirmation of a modified peptide was based on the following criteria: *1*) signal-to-noise (S/N) of all transitions above 10; *2*) ≤20% deviation of relative abundance (*see* Eq. 1) (Geib et al., 2019) for the first transition and ≤30% deviation for second and third transitions) from HP-CAM-modified peptide standard; and *3*) within 60 s of the corresponding HP-CAM peptide’s retention time, to correct for small drifts in RT over the acquisition batch.

$$Relative\;abundance=\frac{A_{X}}{\sum_{i=1}^{3}A_{i}}$$ (1)

APAP-modified peptides with multiple cysteines, containing at least one CAM-cysteine, did not meet the second (or third) criteria, due to no standard samples available for comparison (all cysteines were HP-CAM modified in reference samples). Therefore, confirmation for these modified peptides incorporated into the MRM method was achieved by the absence of corresponding signals in HP-CAM reference samples.

## Results

The goal of this study was to assess the applicability of a targeted LC-MS/MS method for *in vivo* biomonitoring of liver protein adducted by reactive metabolites in multiple samples from rat and mouse, and to compare the results obtained *via* data-dependent high-resolution MS/MS assays. With the aim of identifying low abundant APAP-modified peptides in this highly complex biological matrix, tryptic digests were fractionated by mixed-mode solid-phase extraction. These fractions were subjected to data-dependent high-resolution tandem mass spectrometry (HRMS/MS) analyses as well as targeted MRM assays, and results from these analyses were compared.

### Detection of APAP Protein Targets *via* High-resolution Data-dependent MS/MS

Four liver digests from each species were initially subjected to untargeted high-resolution MS/MS to identify potentially novel target proteins. In total, 15 APAP-modified mouse peptides (from 14 target proteins) were identified (with over 95% peptide confidence). These included five peptides with more than one cysteine, one being APAP-bound and the other(s) carbamidomethylated, however the exact location of the APAP modification was found in each case, based on unique y and b-ions in their high-resolution MS/MS spectra. A specifically challenging example was seen for peptides KPIGLC^174^C^175^IAPVLAAK and C^174^C^175^IAPVLAAK from glutamine amidotransferase-like class 1 domain-containing protein 3A (and its rat ES1 protein homolog) (*see* Supplementary Figure S1), where the APAP modification could be pinpointed on Cys175. From the rat liver digests, 17 distinct peptides were identified as APAP-modified from 16 proteins. Interestingly, the modified peptide found from triosephosphate isomerase in rat, which has two cysteine residues (CLGELICTLNAAK), was detected both as singly and doubly APAP-modified. A summary of all APAP-modified peptides identified from data-dependent experiments can be found in Table 1. From these experiments, several protein orthologs were found in both rodent species, modified at the same cysteine site.

**TABLE 1:** APAP-modified peptides in rat and mouse liver identified by data-dependent high-resolution MS/MS.

Protein ID	Protein Name	Sequence	Conf.	Obs. *m/z*	z
Q9CRB3|HIUH_MOUSE	5-hydroxyisourate hydrolase	LSRLEAPC*QQWMELR	99	670.3344	3
Q8CDE2|CALI_MOUSE	Calicin	IHC*NDFLIK	99	626.3102	2
P16015|CAH3_MOUSE	Carbonic anhydrase 3	EAPFTHFDPSCLFPAC*R	99	715.3168	3
Q64458|CP2C29_MOUSE	Cytochrome P450 2C29	FIDLLPTSLPHAVTC*DIK	99	726.7081	3
Q5MPP0|FA2H_MOUSE	Fatty acid 2-hydroxylase	LAAGAC*WVR	99	1095.5390	1
Q9D172|GAL3A_MOUSE	Glutamine amidotransferase-like class 1 domain-containing protein 3A, mitochondrial	CC*IAPVLAAK	99	597.8044	2
		KPIGLCC*IAPVLAAK	99	568.3172	3
P15105|GLNA_MOUSE	Glutamine synthetase	C*IEEAIDKLSKR	99	389.2080	4
P06151|LDHA_MOUSE	L-lactate dehydrogenase A chain	IVSSKDYC*VTANSK	99	555.2719	3
Q9CXT8|MPPB_MOUSE	Mitochondrial-processing peptidase subunit beta	IVLAAAGGVC*HNELLELAK	99	690.7044	3
Q6PG95|CRML_MOUSE	Protein, cramped-like	KSSQELYGLIC*YGELR	98	1004.4960	2
Q99LX0|PARK7_MOUSE	Protein/nucleic acid deglycase DJ-1	GLIAAIC*AGPTALLAHEVGFGCK	99	806.7496	3
P24549|AL1A1_MOUSE	Retinal dehydrogenase 1	YC*AGWADK	99	531.7211	2
Q63836|SBP2_MOUSE	Selenium-binding protein 2	C*GPGYPTPLEAMK	99	756.8463	2
P17751|TPIS_MOUSE	Triosephosphate isomerase	C*LGELICTLNAANVPAGTEVVCAPPTAYIDFAR	99	914.6931	4
P23457|DIDH_RAT	3-alpha-hydroxysteroid dehydrogenase	SIGVSNFNC*R	99	623.2875	2
P21775|THIKA_RAT	3-ketoacyl-CoA thiolase A, peroxisomal	QC*SSGLQAVANIAGGIR	99	598.6364	3
P16638|ACLY_RAT	ATP-citrate synthase	YIC*TTSAIQNR	99	709.8419	2
Q03248|BUP1_RAT	Beta-ureidopropionase	C*PQIVR	99	432.7230	2
P05179|CP2C7_RAT	Cytochrome P450 2C7	FINFVPTNLPHAVTC*DIK	99	726.7081	3
P07153|RPN1_RAT	Dolichyl-diphosphooligosaccharide--protein glycosyltransferase subunit 1	VAC*ITEQVLTLVNKR	99	612.6727	3
P56571|ES1_RAT	ES1 protein homolog, mitochondrial	CC*IAPVLAAK	99	597.8040	2
P49889/90, P52844|	Estrogen sulfotransferase (Ste2, isoforms 1/3)	NNPC*TNYSMLPETMIDLK	99	745.0038	3
STIE1/2/3_RAT					
O88618|FTCD_RAT	Formimidoyltransferase-cyclodeaminase	TC*ALQEGLR	99	570.2795	2
Q58FK9|KAT3_RAT	Kynurenine-oxoglutarate transaminase 3	ALSC*LYGK	96	502.2493	2
P57113|MAAI_RAT	Maleylacetoacetate isomerase	ALLALEAFQVSHPC*R	99	601.9792	3
O88767|PARK7_RAT	Protein/nucleic acid deglycase DJ-1	GLIAAIC*AGPTALLAHEVGFGCK	99	806.7489	3
P17988|ST1A1_RAT	Sulfotransferase 1A1	MKENC*MTNYTTIPTEIMDHNVSPFMR	99	813.8645	4
P11232|THIO_RAT	Thioredoxin	C*MPTFQFYK	99	657.2881	2
P48500|TPIS_RAT	Triosephosphate isomerase	C*LGELICTLNAAK	99	777.8861	2
		C*LGELIC*TLNAAK	99	823.8993	2
P09118|URIC_RAT	Uricase	SIETFAMNIC*EHFLSSFSHVTR	99	677.0681	4

* APAP modification site.

### Building HP-CAM-Peptide Standard Library

**FIGURE 1:**
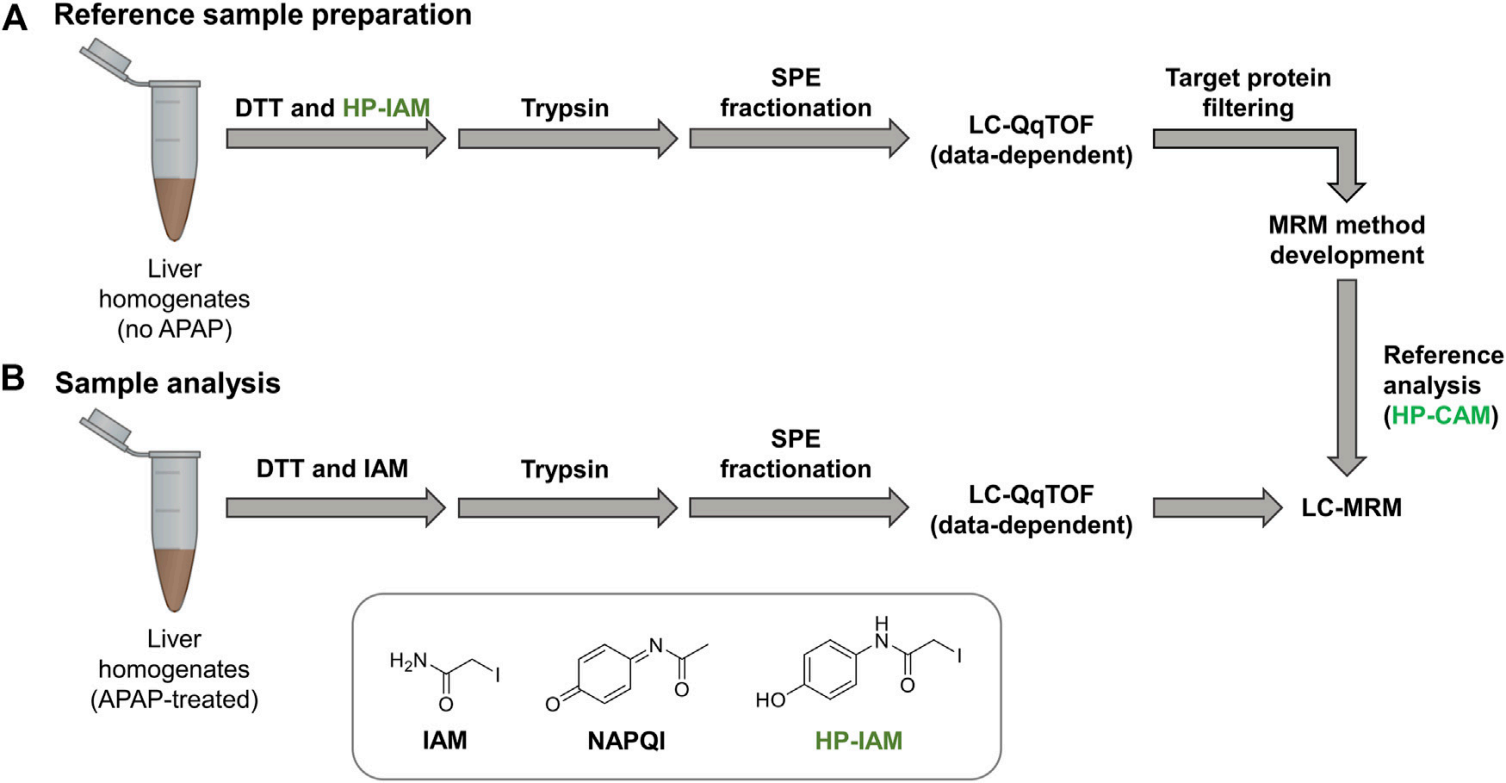
Reference samples **(A)** were prepared using non-treated mouse and rat liver homogenates and HP-IAM as the cysteine alkylation reagent. Modified peptides of interest from potential protein targets were selected for MRM method preparation. Final LC-sMRM methods were used to screen for modified peptides in **(B)** APAP-treated samples. Data-dependent high-resolution MS/MS experiments were performed on reference samples as well as selected APAP-treated rat and mouse samples for identifying modified peptides *via* database searching with custom modification (+C_8_H_7_NO_2_ addition onto Cys residues).

Control liver samples were alkylated with hydroxyphenyl iodoacetamide (HP-IAM) prior to trypsin digestion, SPE fractionation and data-dependent LC-MS/MS analysis (*see* Figure 1). HP-CAM modification yields a positional isomer of APAP-modification on all cysteines and modified peptides were identified using the same criteria as APAP-modified peptides from database searching. In total, 1466 HP-CAM-modified proteins with 4554 distinct HP-CAM peptides were identified in the mouse control sample; and 1073 HP-CAM-modified proteins with 3599 distinct HP-CAM-peptides were found in the rat control sample. The resulting list of HP-CAM peptides was further filtered to include peptides of a select list of potential protein targets. These rat and mouse protein targets were based on our data-dependent results, as well as previous APAP *in vitro* binding studies (Golizeh et al., 2015; Geib et al., 2019), and additional APAP protein targets found in the Target Protein Database (TPDB) (Hanzlik et al., 2020). The resulting filtered HP-CAM-peptide list contained information on which SPE fraction contained the identified peptide, as well as peptide retention time, charge state, and its three most intense fragment ions (preferably with fragment *m/z* > precursor *m/z*). These target peptides selected for MRM analysis are in Supplementary Table S3.

Scheduled MRM methods were built, with three transitions per peptide, and separated based on species and SPE fraction. MRM transitions of modified peptides were also monitored in adjacent fractions as those where they were detected in HP-CAM samples, since peptides can often elute in more than one fraction. In the case of two peptides from mouse glutamine amidotransferase and rat ES1 protein homolog, where one Cys was APAP-modified and the other was carbamidomethylated, retention times and fragment ions from data-dependent MS/MS were used to build the MRM method. Unfortunately, several other peptides with multiple cysteines were not monitored by MRM as their signals were not confirmed when developing the targeted method.

### Detection of APAP-Peptides *via* LC-MRM

**FIGURE 2:**
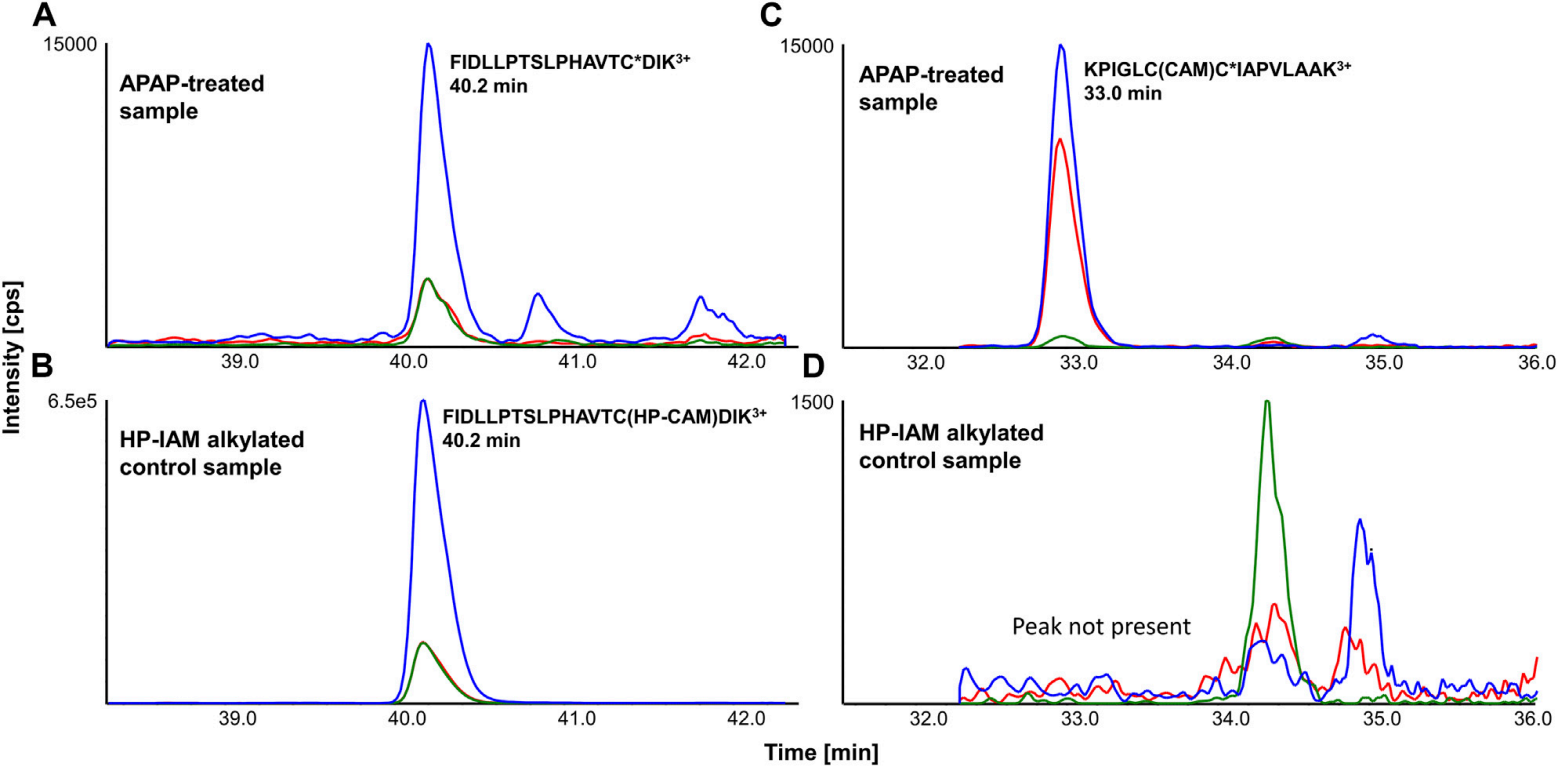
Representative LC-MRM chromatograms of **(A)** FIDLLPTSLPHAVTC*DIK^3+^ (from cytochrome P450 2C29) from an APAP-treated liver digest and **(B)** its reference in HP-CAM control sample in mouse (from SPE fraction 3), as well as **(C)** KPIGLCC*IAPVLAAK^3+^ (from mitochondrial ES1 protein homolog) from an APAP-treated liver digest and **(D)** its correspondingly absent signal in the HP-CAM control sample in rat due to the presence of two cysteines in its sequence (from SPE fraction 8). The star (*) in the peptide sequence indicates the confirmed site of APAP modification.

APAP-treated and HP-CAM alkylated liver digests were analyzed by scheduled LC-MRM methods specific to each SPE fraction. LC-MRM peaks were integrated, and relative peak areas for each transition (transition/sum of all transitions) as well as retention times were compared with HP-CAM signals for the same peptide. Figure 2 shows a representative example of a confirmed target LC-MRM for a mouse CYP 2C29 peptide, found *via* data-dependent MS/MS and confirmed by MRM analyses. The analogous peptide in rat CYP2C7 was also confirmed *via* data-dependent MS/MS and LC-MRM. Figure 2 also shows the example of the tryptic peptide with two cysteines, but only one of them being APAP-modified, from mouse mitochondrial glutamine-amidotransferase-like 1 domain-containing protein 3A and rat mitochondrial ES1 protein homolog. Remarkably, the non-tryptic peptide containing the same modified cysteine was found in our data-dependent analyses in both species, as well as screened for during LC-MRM analyses, with the same modification site being confirmed in all liver samples. These modified peptides were confirmed based on the absence of a corresponding signal in HP-CAM-control samples.

From eight APAP-treated mouse livers (two animal each at 150 and 300 mg/kg, either 2 and 6 h post dose), LC-MRM confirmed 13 distinct APAP-modified sites from 13 different proteins. From Table 2, the distribution of these confirmed targets is shown for each dosing regime, with many of them being confirmed in all samples, and five being detectable only at the 300 mg/kg dosing level. The two dosing levels at 150 and 300 mg/kg in mouse were chosen since this species is known to be more susceptible to APAP-related hepatotoxicity, compared to rat which could tolerate the higher dose at 600 mg/kg. From previous work, it was found that at 600 mg/kg dose in rat, APAP-albumin adducts did not decrease over the 24 h (LeBlanc et al., 2014), however, shorter timepoints (2 and 6 h) were chosen for mouse since they exhibit higher toxicity.

**TABLE 2:** APAP-modified peptides in mouse liver identified *via* LC-MRM.

Acc. #	Protein name	Cys site	Peptide sequence*	z	# Of hits (*n* = 2 each) (mg/kg, time)			
					150/2 h	300/2 h	150/6 h	300/6 h
Q91V92	ATP-citrate synthase	C20	YIC*TTSAIQNR	2	2	2	2	2
O35490	Betaine-homocysteine *S*-methyltransferase 1	C131	QVADEGDALVAGGVSQTPSYLSC*K	3	0	1	0	2
Q64458	Cytochrome P450 2C29	C372	FIDLLPTSLPHAVTC * DIK	3	2	2	2	2
Q9D172	Glutamine amidotransferase-like 1 domain-containing protein 3A, mitochondrial	C175	KPIGLC(CAM)C * IAPVLAAK ^a^	3	2	2	2	1
Q9D172	Glutamine amidotransferase-like 1 domain-containing protein 3A, mitochondrial	C175	C(CAM)C * IAPVLAAK ^a^	2	2	2	2	2
P15105	Glutamine synthetase	C269	C * IEEAIDK	2	1	2	2	2
P16858	Glyceraldehyde-3-phosphate dehydrogenase	C22	AAIC*SGK	2	0	0	0	2
P17563	Methanethiol oxidase	C8	C*GPGYSTPLEAMK	2	0	2	1	2
Q91VS7	Microsomal glutathione *S*-transferase 1	C50	VFANPEDC*AGFGK	2	0	1	0	0
Q9CXT8	Mitochondrial-processing peptidase, beta	C248	IVLAAAGGVC * HNELLELAK	3	2	2	2	2
P24549	Retinal dehydrogenase 1	C133	YC*AGWADK	2	1	0	0	1
Q63836	Selenium-binding protein 2	C8	C * GPGYPTPLEAMK	2	0	2	0	1
Q64442	Sorbitol dehydrogenase	C106	EVDEYC*K	2	0	0	0	1
P10639	Thioredoxin	C73	C*MPTFQFYK	2	0	2	2	1

* APAP modification site. Underlined peptides (or modification sites) were also identified in data-dependent MS/MS experiments.

a Identified based on an absent signal in the reference (HP-CAM) sample.

From the analysis of four rat liver samples, 23 distinct APAP-peptides were confirmed, from 21 different proteins (Table 3). Two proteins were close homologs, CYP 2C6 and 2C7, with very similar peptide sequences, though the 2C6 peptide was found *via* LC-MRM only in one animal, compared to the 2C7 modified peptide being well detected in all rat livers. Two peptides with the same modification site were found for the ES1 protein homolog in all rats. Methanethiol oxidase also had two modified cysteine sites, though one was detectable in only one rat liver, whereas the one modified at Cys8 was detected in all rat livers, as well as in five of eight mice, including all livers at the 300 mg/kg dose. Also in mouse, a very similar protein, selenium-binding protein 2, was modified at the same cysteine site, with a peptide differing only by one amino acid, in three of four mice at the higher dose. Another interesting example is that of triosephosphate isomerase, which was found to have one peptide having two APAP-modified cysteines by data-dependent analyses. LC-MRM results confirmed this doubly modified peptide in all four rat livers. As shown in Table 1, the same peptide with only one cysteine modified was also identified from rat as well as the singly modified mouse peptide, however these peptides were not screened for by LC-MRM. The mouse peptide specifically was a very long peptide with 33 residues and a charge state of 4+, making it quite difficult to screen for *via* LC-MRM without a corresponding signal in the HP-CAM reference sample. Another example of a target protein found in both species *via* data-dependent HRMS/MS analyses only is protein/nucleic acid deglycase DJ-1 (also known also Parkinson disease protein 7 homolog), with an identical peptide in mouse and rat incorporating two cysteines, one of which was APAP-modified. This large peptide was not easily amenable to MRM analysis due to the lack of significantly intense fragment ions, as well as no appropriate HP-CAM peptide for method optimisation. LC-MRM data with integrated peak areas and retention times from all samples have been made available in Supplementary Table S4.

**TABLE 3:** Identified APAP-modified peptides in rat liver *via* LC-MRM.

Acc. #	Protein name	Cys site	Peptide*	z	# Of hits
					600 mg/kg (*n* = 4)
P23457	3-α-hydroxysteroid dehydrogenase	C170	SIGVSNFNC * R	2	4
P16638	ATP-citrate synthase	C20	YIC * TTSAIQNR	2	4
O09171	Betaine-homocysteine *S*-methyltransferase 1	C131	QVADEGDALVAGGVSQTPSYLSC*K	3	4
P05178	Cytochrome P450 2C6	C372	FIDLIPTNLPHAVTC*DIK	3	1
P05179	Cytochrome P450 2C7	C372	FINFVPTNLPHAVTC * DIK	3	4
P36365	Dimethylaniline monooxygenase [N-oxide-forming] 1	C35	SC * DLGGLWR ^a^	2	4
P07153	Dolichyl-diphosphooligosaccharide protein glycosyltransferase subunit 1	C475	VAC * ITEQVLTLVNKR	3	4
P49889/90	Estrogen sulfotransferase 1/2/3	C237	NNPC * TNYSMLPETMIDLK	3	3
P52844					
P56571	ES1 protein homolog, mitochondrial	C175	C(CAM)C * IAPVLAAK	2	4
P56571	ES1 protein homolog, mitochondrial	C175	KPIGLC(CAM)C*IAPVLAAK	3	4
O88618	Formimidoyltransferase-cyclodeaminase	C438	TC * ALQEGLR	2	3
P09606	Glutamine synthetase	C269	C*IEEAIDK	2	4
P57113	Maleylacetoacetate isomerase	C205	ALLALEAFQVSHPC * R	3	4
Q8VIF7	Methanethiol oxidase	C371	GGSVQVLEDQELTC*QPEPLVVK	3	1
Q8VIF7	Methanethiol oxidase	C8	C*GPGYATPLEAMK	2	4
P08011	Microsomal glutathione *S*-transferase 1	C50	VFANPEDC*AGFGK	2	2
Q03346	Mitochondrial-processing peptidase subunit beta	C248	IVLAAAGGVC*HNELLELAK	3	3
Q63716	Peroxiredoxin-1	C173	HGEVC*PAGWKPGSDTIKPDVNK	4	4
P11598	Protein disulfide-isomerase A3	C244	FIQESIFGLC*PHMTEDNK	3	3
P17988	Sulfotransferase 1A1	C232	MKENC * MTNYTTIPTEIMDHNVSPFMR	4	1
P48500	Triosephosphate isomerase	C21/27	C * LGELIC * TLNAAK	2	4
P11232	Thioredoxin	C73	C * MPTFQFYK	2	4
P09118	Uricase	C95	SIEFAMNIC * EHFLSSFSHVTR	4	2

* APAP modification site. Underlined peptides were also identified in data-dependent MS/MS experiments (^a^with inclusion list).

### Inclusion List High-resolution MS/MS for APAP-Modified Peptides

All APAP-modified peptides detected *via* LC-MRM not detected by data-dependent analyses were screened a second time by incorporating an inclusion list for targeted precursor ions. The same fractions with confirmed LC-MRM hits were analyzed. Only one modified peptide was ultimately detected with sufficient spectral confidence >95% (*see* high-resolution MS/MS in Supplementary Figure S1) for the modified peptide from rat dimethylaniline (*N*-oxide forming) monooxygenase, which was confirmed in all four rat livers by LC-MRM. This protein had been added to the LC-MRM method due to its presence as an APAP target in TPDB (Leeming et al., 2017). The same modification site that was previously found was confirmed.

## Discussion

### Comparison of Targeted and Untargeted Workflows

Targeted LC-MS/MS workflows were specifically designed to utilize the high duty cycle and low limit of detection of multiple reaction monitoring (MRM). The detection of modified peptides was optimized using scheduled MRM methods built for each SPE fraction for a given species separately. By using HP-IAM in the reference samples, it was possible to mimic APAP-derived covalent modification on cysteines in the final protein digest. HP-CAM peptides were used to confirm which fractions to monitor for each modified peptide, as well as LC retention time and MS/MS fragmentation behavior, with relatively high signal intensity. Whenever possible, peptide candidates in one species were translated to the closest protein ortholog in the other species. Modified peptides not amenable to MRM transition development were omitted in the final scheduled MRM method, based on giving an appropriate signal for three MRM transitions in reference samples.

In general, targeted LC-MRM analyses showed far superior detectability of APAP-modified peptides in these samples. An important advantage that MRM detection has over data-dependent MS/MS, is that each modified peptide signal is continuously monitored, instead of depending on the automatic selection of a precursor of interest for MS/MS acquisition, which leads unfortunately to much less reproducible data as well as the loss of low-abundant peptides in highly complex samples. For this reason, many modified peptides would not have a chance of being identified with conventional untargeted bottom-up proteomics workflows. An important caveat to the MRM method, however, is that method development is more time-consuming, and in the case of APAP-modified peptides for this study, the custom alkylation HP-CAM peptides were crucial for confirmation. Also, MRM sensitivity depends highly on the fragmentation behavior of the ionized peptide. Certain highly charged or large peptides necessitate more optimization by changing collision energies and selected multiple fragment ions, and without an appropriate standard, this is impossible. High-resolution DDA experiments are more flexible, for example for those with multiple cysteines with only one being modified by APAP. A unique opportunity was afforded to specifically design LC-MRM assays for confirming protein targets by the possibility of having a positional isomer of APAP quantitatively modifying all cysteines in the liver homogenates. Without this standard as a reference, these hits may have been detected but not as easily confirmed.

### Confirmed APAP Protein Targets

**FIGURE 3:**
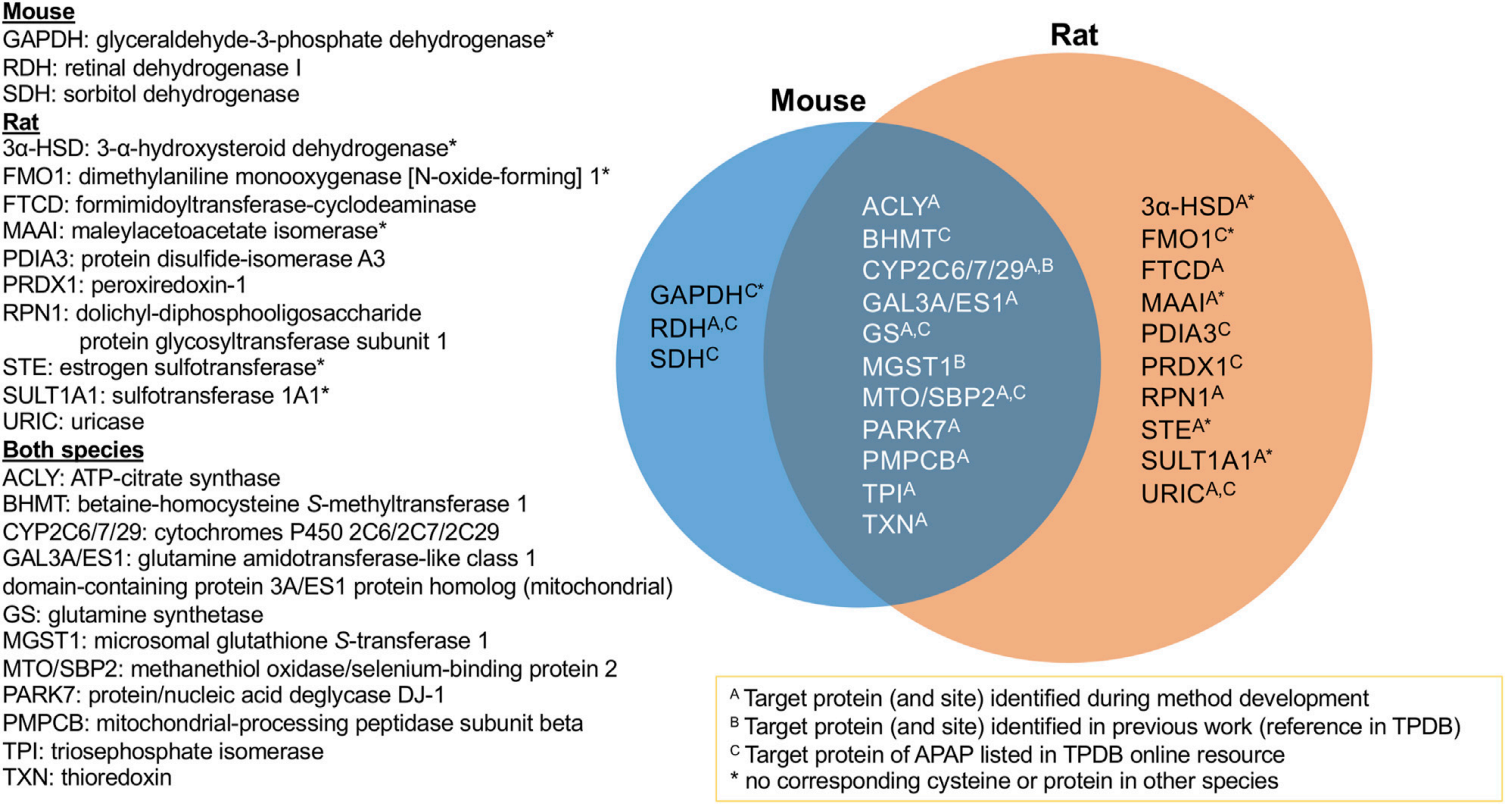
Summary of APAP protein targets confirmed in rat and mouse liver samples *via* targeted LC-MRM analyses.

The aim of this study was to identify protein targets of APAP’s reactive metabolite in mouse and rat liver to help better understand the mechanisms of APAP-induced hepatotoxicity. Using a combination of high-resolution data-dependent MS/MS and scheduled MRM experiments, a multitude of targets have been found for the first time, as well as confirming others previously reported targets of APAP from literature. It was possible to compare protein targets and modification sites in mouse and rat livers. Figure 3 summarizes the proteins confirmed in this study, with those overlapping between both species. All those listed were confirmed by LC-MRM analyses, except protein/nucleic acid deglycase DJ-1, which was found by data-dependent analyses in both species, however the modified peptide was not amenable to MRM analysis. What was quite striking was that for all proteins common to both species, the same modification sites were confirmed. This suggests the selective modification from APAP on these proteins, some of which could be specifically involved in the mechanism of drug-induced liver injury. Of the 24 protein groups listed in Figure 3, 17 were initially identified by DDA experiments and 12 were listed as APAP targets in TPDB. Though not reported as APAP targets, a few were also noted in TPDB as targets of other xenobiotics.

From the 10 protein targets confirmed in rat only, four of these did not have corresponding cysteine residues in the mouse ortholog, namely two sulfotransferases, dimethylaniline monooxygenase and maleylacetoacetate isomerase. One other protein, 3-alpha hydroxysteroid dehydrogenase (3α-HSD), does not have a corresponding ortholog in mouse at all. In the case of the three targets found uniquely in mouse, one protein has no corresponding cysteine in the rat protein (GAPDH). Retinal dehydrogenase, though the same peptide was monitored for both species, was only found in two of the eight mouse samples. As for sorbitol dehydrogenase, the corresponding ortholog peptide was not incorporated into the MRM method, since it was not found in the rat HP-CAM reference sample and thus could not be optimized in the targeted method.

One example of an APAP target found in rat, with no corresponding cysteine in the mouse ortholog, was estrogen sulfotransferase (EST), a cytosolic enzyme that inhibits estrogen activity by conjugating a sulfonate group to it (Strott, 1996). The APAP-modified peptide NNPC^237^TNYSMLPETMIDLK, which is common to rat estrogen sulfotransferase isoforms 1, two or 3, was identified by data-dependent experiments, as well as confirmed by LC-MRM. Similarly, modified rat sulfotransferase 1A1 was found from data-dependant experiments, and was also confirmed by LC-MRM.

3α-HSD catalyzes NAD(P)^+^-dependent oxidoreduction of steroids and dihydrodiols. Multiple forms of 3α-HSD have been identified in rat liver with a role in xenobiotic metabolism and intracellular transport of bile acids (Turley and Dietschy, 1978). In rat liver, 3α-HSD can metabolize polycyclic aromatic hydrocarbon carcinogens and also affects net bile acid transport across hepatocytes (Dufort et al., 2001). Although not previously found to be a target of APAP binding, 3α-HSD has been identified as a target of bromobenzene (Koen et al., 2007), thiobenzamide (Ikehata et al., 2008) and furan (Moro et al., 2012), without site specificity noted. From this study, Cys170 was confirmed as the APAP modification site.

All 11 protein groups confirmed in both species (Figure 3) had the same cysteine modification sites. ATP-citrate synthase (ACLY) was found to be a target of APAP in both species by LC-MRM, following initial identification of the modified rat peptide *via* data-dependent MS/MS. The modified residue Cys20 is in its ATP-binding domain, which catalyses the conversion of citrate to acetyl-CoA in the TCA cycle. Glutamine synthetase (GS) is a cytosolic enzyme, also associated to mitochondria and ER. GS was previously identified as an APAP target by immunochemical detection in mice (Bulera et al., 1995). Its enzymatic activity was also significantly decreased by APAP in cultured hepatocytes. After identifying the Cys269 modification site by data-dependent MS/MS in mouse, the APAP peptide was confirmed in both species by LC-MRM. Triosephosphate isomerase (TPI) is a glycolytic enzyme necessary for energy production. Cytoplasmic TPI has been reported as a covalent target of bromobenzene (Koen et al., 2007), mycophenolic acid (Asif et al., 2007), diaminochlorotriazine (Dooley et al., 2008), 4-bromophenol (Koen et al., 2012), tienilic acid (Methogo et al., 2007) and thioacetamide (Koen et al., 2013). Although TPI was only confirmed by LC-MRM for the doubly APAP-modified peptide in rat, it was also found to be singly modified at the same site in both species *via* data-dependent experiments. It, therefore, was classified as a target of APAP in both species.

Rat ES1 protein homolog and mouse glutamine amidotransferase-like class 1 domain-containing protein 3A are orthologous mitochondrial proteins confirmed as modified in both species from these results. ES1 was found to be elevated in Down syndrome, potentially as an antioxidant response to increased mitochondrial ROS production (Shin et al., 2004). Other mitochondrial protein targets confirmed in both species were mitochondrial-processing peptidase subunit beta (PMPCB), responsible for the proteolytic processing of mitochondrially encoded precursor polypeptides, and protein/nucleic acid deglycase DJ-1, also known as Parkinson disease protein 7 homolog, PARK7. PARK7 catalyzes the deglycation of Maillard adducts between proteins or DNA and reactive carbonyls (Richarme et al., 2015; Richarme et al., 2017) and protects cells from H_2_O_2_-related death (Sekito et al., 2006). It is required for correct mitochondrial morphology and function. The same modification site (Cys106) of PARK7 was recently found following APAP treatment of liver microtissues using data-independent MS/MS acquisition (Bruderer et al., 2015). Mitochondrial protein targets are of specific interest, as they have been correlated to the initiation of APAP’s hepatotoxicity (Tirmenstein and Nelson, 1989; Ramachandran and Jaeschke, 2019). They have been proposed as a source of APAP-induced mitochondrial dysfunction (Hu et al., 2016), and are believed to be linked to the inhibition of electron transport chain (Lee et al., 2015), reactive oxygen species formation (Jiang et al., 2015) and mitogen-activated protein kinase and c-Jun N terminal kinase activation (Nguyen et al., 2021).

Two protein targets found previously by our group *in vitro* in rat liver microsomes, CYP2C6 and MGST1, were also confirmed here. Therefore, further *in vitro* work could be of value for finding biologically relevant targets in other cellular compartments, such as the cytosol and mitochondria. MGST1 had a common APAP-modified peptide in both species confirmed by LC-MRM. Orthologs CYP2C6 and 2C7 in rat, and 2C29 in mouse, were all found to be modified at Cys372 by APAP from data-dependent and MRM experiments. The latter two were consistently found as positive hits by LC-MRM in all animals of their respective species.

Other protein targets common to both species included betaine-homocysteine *S*-methyltransferase 1 (BHMT), methanethiol oxidase (MTO)/selenium binding protein 2 (SBP2) and thioredoxin (TXN). BHMT catalyzes betaine conversion to homocysteine in the biosynthesis of dimethylglycine and methionine. BHMT deficiency has been linked to hepatocellular carcinoma and fatty liver disease (Teng et al., 2011). BHMT was monitored as a potential target due to its presence in the TPDB list of APAP protein targets (Leeming et al., 2017), and confirmed the previously elucidated modification site. MTO and SBP2 are selenium-binding proteins, previously reported as major APAP targets in the liver (Bartolone et al., 1992; Pumford et al., 1992; Qiu et al., 1998) as well as other xenobiotics (Shipkova et al., 2004; Koen et al., 2007; Meier et al., 2007; Koen et al., 2012). Involved in organo-sulfur degradation, these proteins may be involved in the sensing of reactive xenobiotics in the cytoplasm (Pol et al., 2018).

Thioredoxin is involved in redox signalling through oxidation of its thiols. The APAP modification site was confirmed on Cys73, the only available free thiol in fully oxidized THX, which is also known to serve as a donor for nitrosylation of proteins under NO stress (Mitchell and Marletta, 2005). APAP overdose-linked oxidation of TXN2 has been previously observed (Ramachandran et al., 2015). Mitochondrial TXN2 and cytosolic TXN1 have been shown to be irreversibly modified by APAP (Jan et al., 2014). APAP-induced oxidation of cytosolic TXN1 results in the dissociation of TXN1 and its binding partner, apoptosis signal-regulating kinase 1 (Nakagawa et al., 2008). A protective effect of administration of albumin-fused recombinant TXN1, 4 h after APAP administration, has also been reported (Tanaka et al., 2014). Interestingly, this is the first report of APAP covalent binding to TXN, though it has been found as a target of other reactive metabolites (Koen et al., 2007; Ikehata et al., 2008; Moro et al., 2012; Koen et al., 2013).

Reactive metabolites can covalently bind to proteins causing downstream immune reactions and/or cell damage (Poprac et al., 2017). APAP protein adduction has long been studied and serves as a model of drug-induced hepatotoxicity, however, its mechanism of action is not yet fully understood (Heard et al., 2016). Results from this study confirmed many previously reported protein targets, as well as their specific modification sites, and adding several new *in vivo* protein targets in two rodent models. These findings can be further investigated by studying protein and/or site-specific effects and mechanisms related to drug-induced toxicity. Mitochondrial protein targets of APAP would be of specific interest for subsequent research since there is already much evidence of mitochondrial signalling being involved in APAP hepatotoxicity.

## Conclusion

An analytical workflow was developed and applied to rat and mouse liver homogenates to investigate protein covalent binding following APAP administration. Using a combination of high-resolution MS/MS and scheduled MRM assays for proteomic analyses and a custom alkylation reagent, many *in vivo* protein targets and their modification sites in both species have been confirmed or newly identified. The protein targets identified could serve to better understand the reactivity of NAPQI *in vivo* and could point towards specific targets of interest linked to acetaminophen-related hepatotoxicity. The performance of targeted scheduled LC-MRM has been demonstrated, based on a curated list of potential candidates, as a powerful tool in LC-MS/MS proteomics of low-abundant modified peptides in highly complex biological samples.

### Associated Content

#### Supporting Information

MRM transitions and retention time settings used for LC-sMRM analyses of individual SPE fractions (two to eight) from rat and mouse digests (Supplementary Tables S1, S2, respectively). Overview of modified peptides screened for in scheduled MRM experiments (Supplementary Table S3). Results from scheduled MRM experiments, including peak areas for each transition monitored, relative areas, and retention times and calculated deviations to the control HP-CAM peptides (Supplementary Table S4). High-resolution MS/MS spectra for APAP-modified peptides identified using data-dependent acquisition (Supplementary Figure S1).

## Data Availability

The datasets presented in this study can be found in online repositories. The names of the repository/repositories and accession number(s) can be found below: ProteomeXchange PRIDE repository, accession no: PXD027674.

## Abbreviations

ACN, acetonitrile; APAP, acetaminophen; BHMT, betaine—homocysteine S-methyltransferase 1; CA3, carbonic anhydrase 3; CAM, carbamidomethylation; CPS1, mitochondrial carbamoyl-phosphate synthase [ammonia]; DTT, dithiothreitol; ER, endoplasmic reticulum; EST, estrogen sulfotransferase; GS, glutamine synthetase; HDAg, hepatitis delta antigen; HP-CAM, N-(4-hydroxyphenyl)-2-carbamidomethylation; HP-IAM, N-(4-hydroxyphenyl)-2-iodoacetamide; 3α-HSD, 3-alpha-hydroxysteroid dehydrogenase; IAM, iodoacetamide; IDA, information-dependent acquisition; IP, intraperitoneal; LC-MS/MS, liquid chromatography-tandem mass spectrometry; MeOH, methanol; MGST1, microsomal glutathione S-transferase 1; MRM, multiple reaction monitoring; MTO, methanethiol oxidase; NAPQI, N-acetyl p-benzoquinone imine; PMPCB, mitochondrial-processing peptidase subunit beta; SDS, n-dodecyl sulfate; S/N, signal-to-noise; SPE, solid-phase extraction; TPDB, Target Protein Database; TPI, triosephosphate isomerase; TXN, thioredoxin.
