# Supplementary material for: Protein Targets of Acetaminophen Covalent Binding in Rat and Mouse Liver Studied by LC-MS/MS
Source: Front Chem. 2021 Aug 20;9:736788. doi: 10.3389/fchem.2021.736788 (PMC8417805; doi:10.3389/fchem.2021.736788)
Supplement: Supplementary file 3 [file Presentation1.PPTX]

## Slide 1
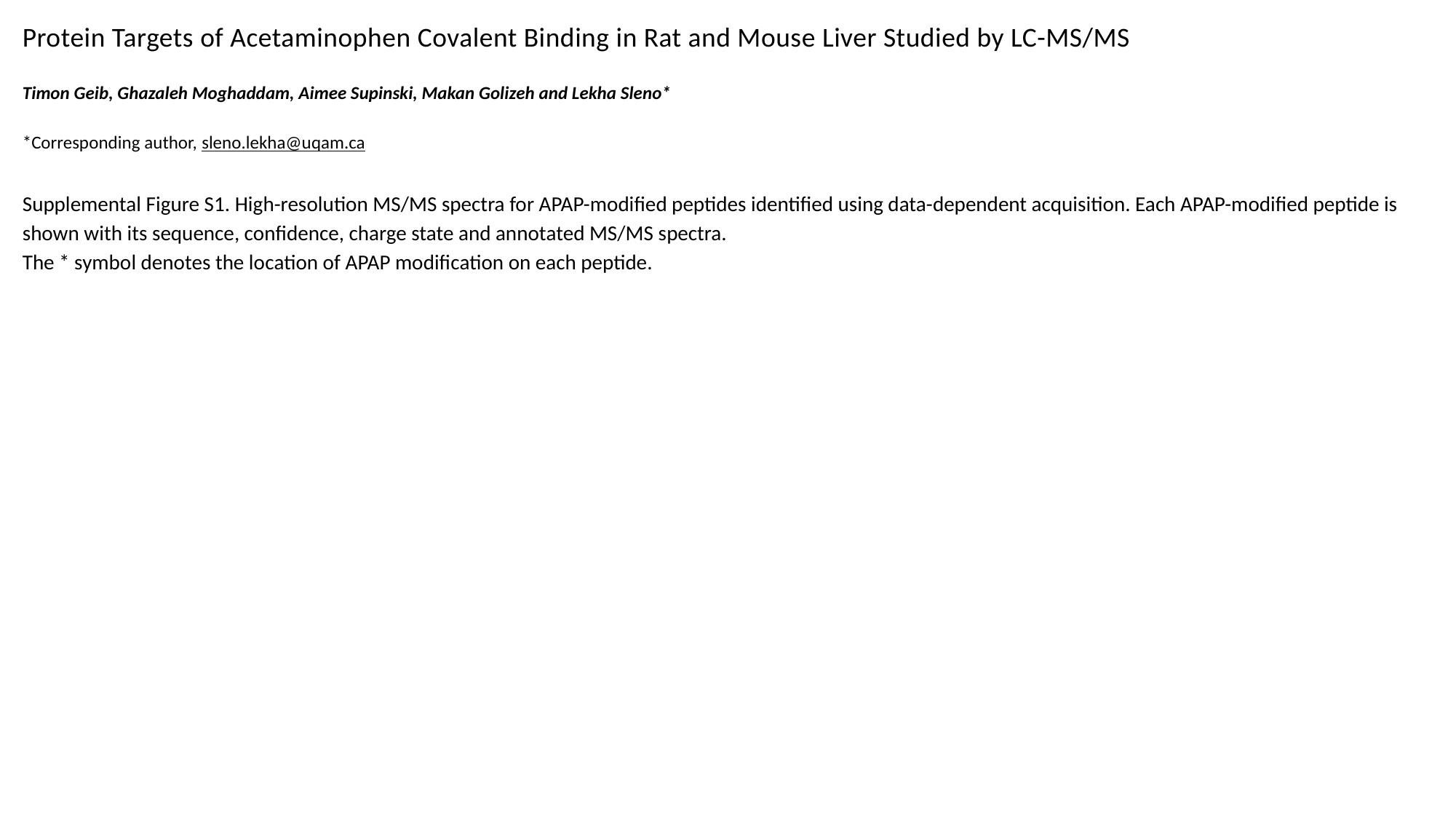

# Protein Targets of Acetaminophen Covalent Binding in Rat and Mouse Liver Studied by LC-MS/MS Timon Geib, Ghazaleh Moghaddam, Aimee Supinski, Makan Golizeh and Lekha Sleno* *Corresponding author, sleno.lekha@uqam.ca Supplemental Figure S1. High-resolution MS/MS spectra for APAP-modified peptides identified using data-dependent acquisition. Each APAP-modified peptide is shown with its sequence, confidence, charge state and annotated MS/MS spectra. The * symbol denotes the location of APAP modification on each peptide.

## Slide 2
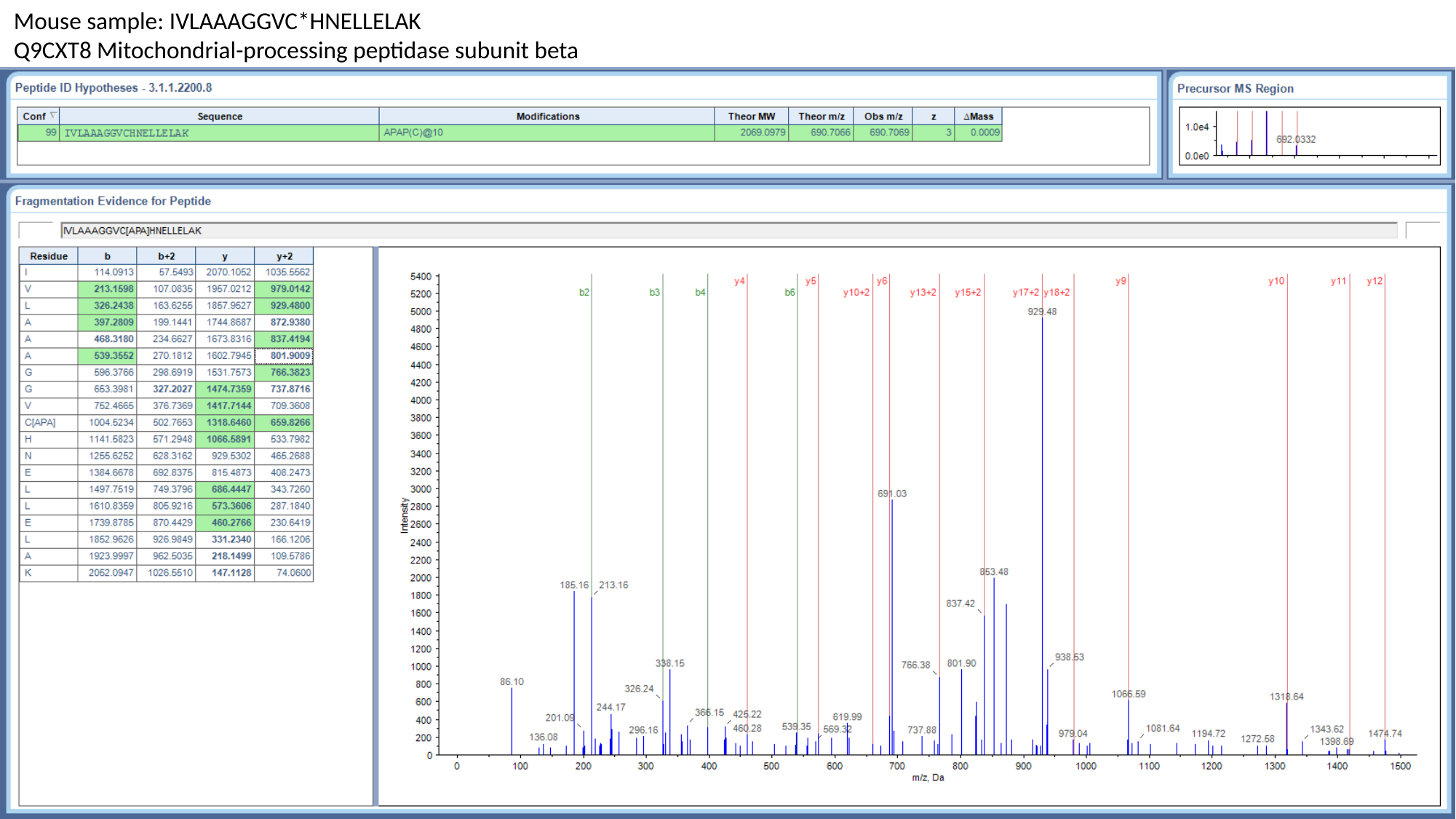

Mouse sample: IVLAAAGGVC*HNELLELAK
Q9CXT8 Mitochondrial-processing peptidase subunit beta

## Slide 3
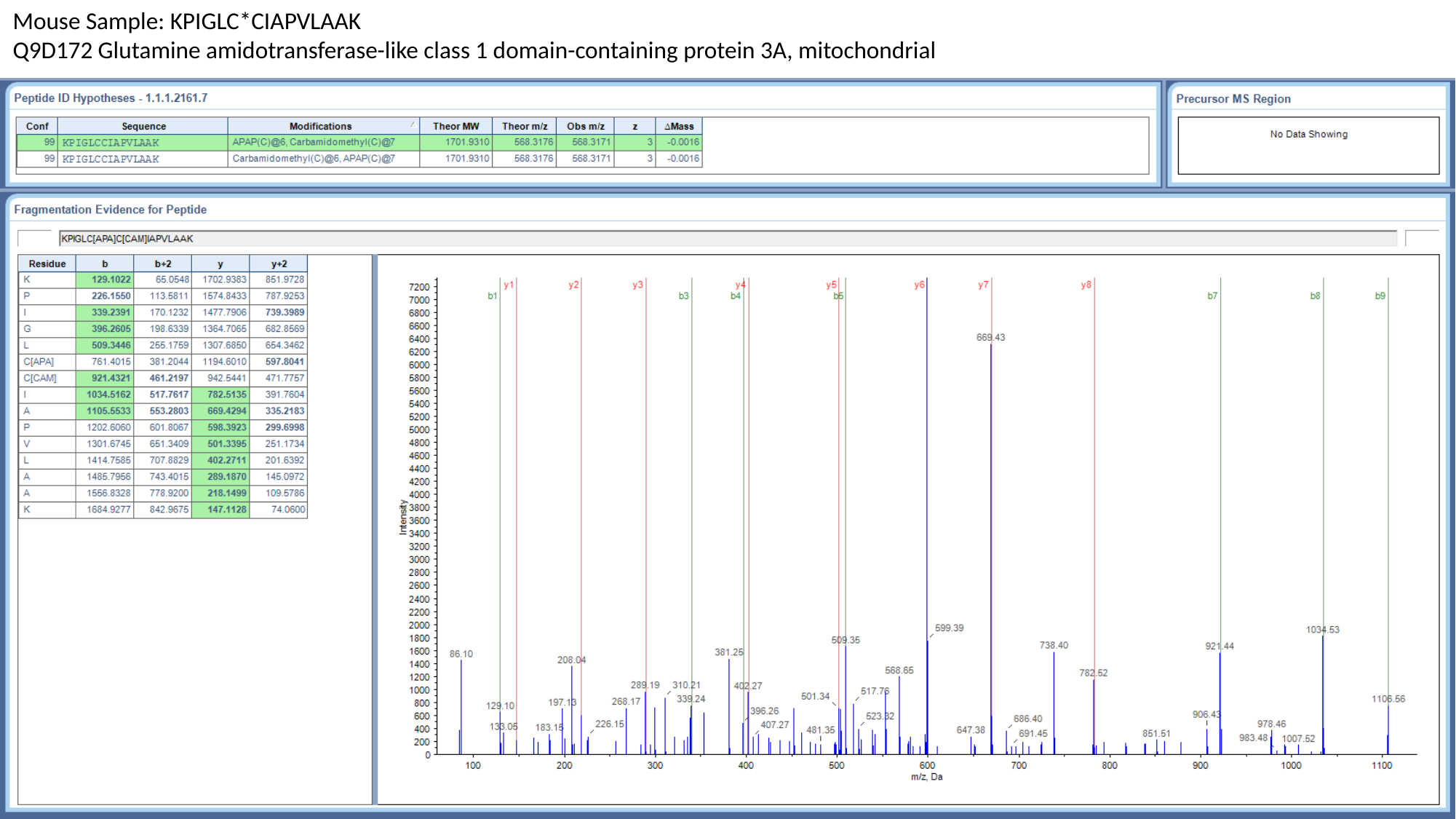

Mouse Sample: KPIGLC*CIAPVLAAK
Q9D172 Glutamine amidotransferase-like class 1 domain-containing protein 3A, mitochondrial

## Slide 4
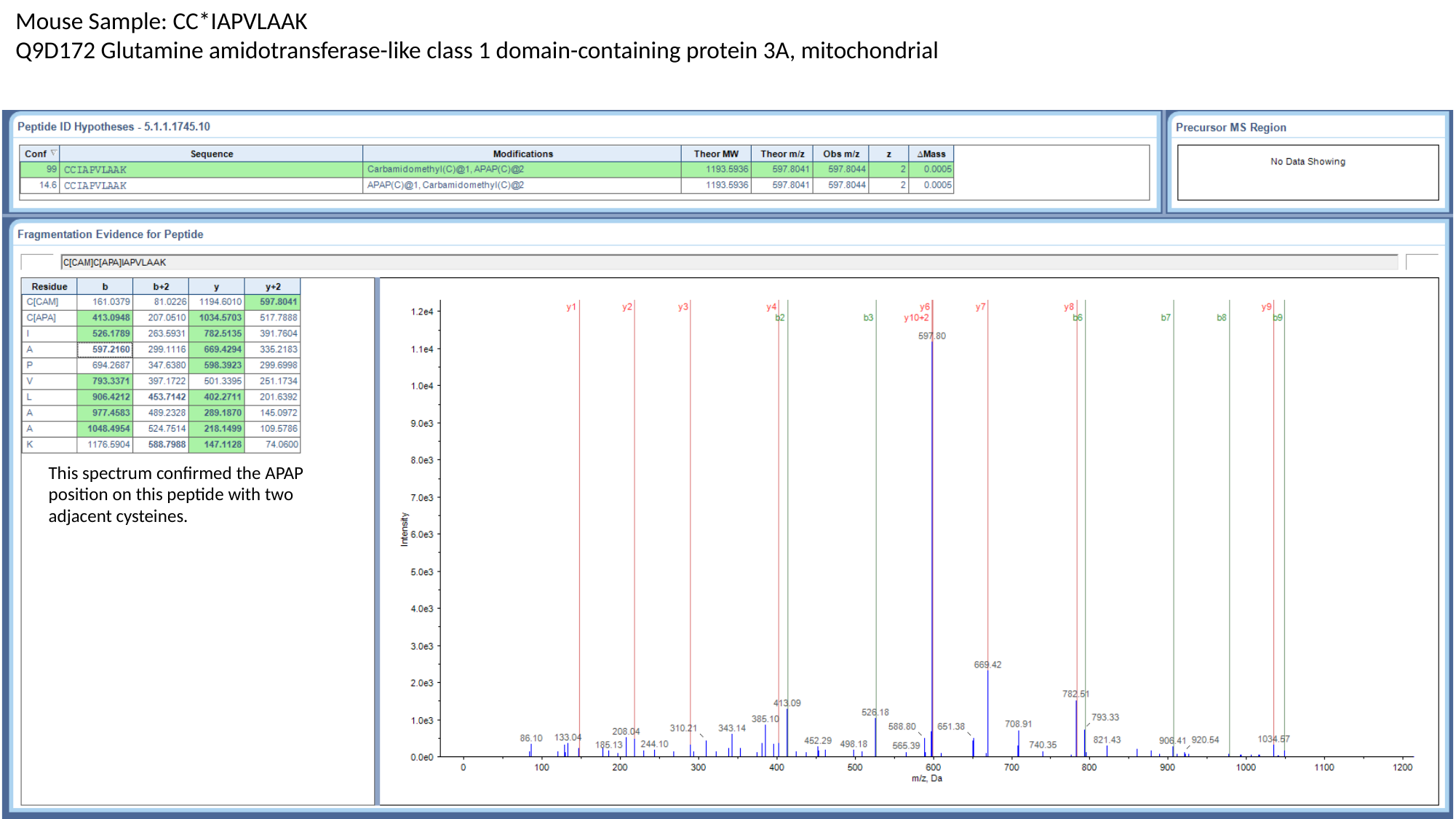

Mouse Sample: CC*IAPVLAAK
Q9D172 Glutamine amidotransferase-like class 1 domain-containing protein 3A, mitochondrial
This spectrum confirmed the APAP position on this peptide with two adjacent cysteines.

## Slide 5
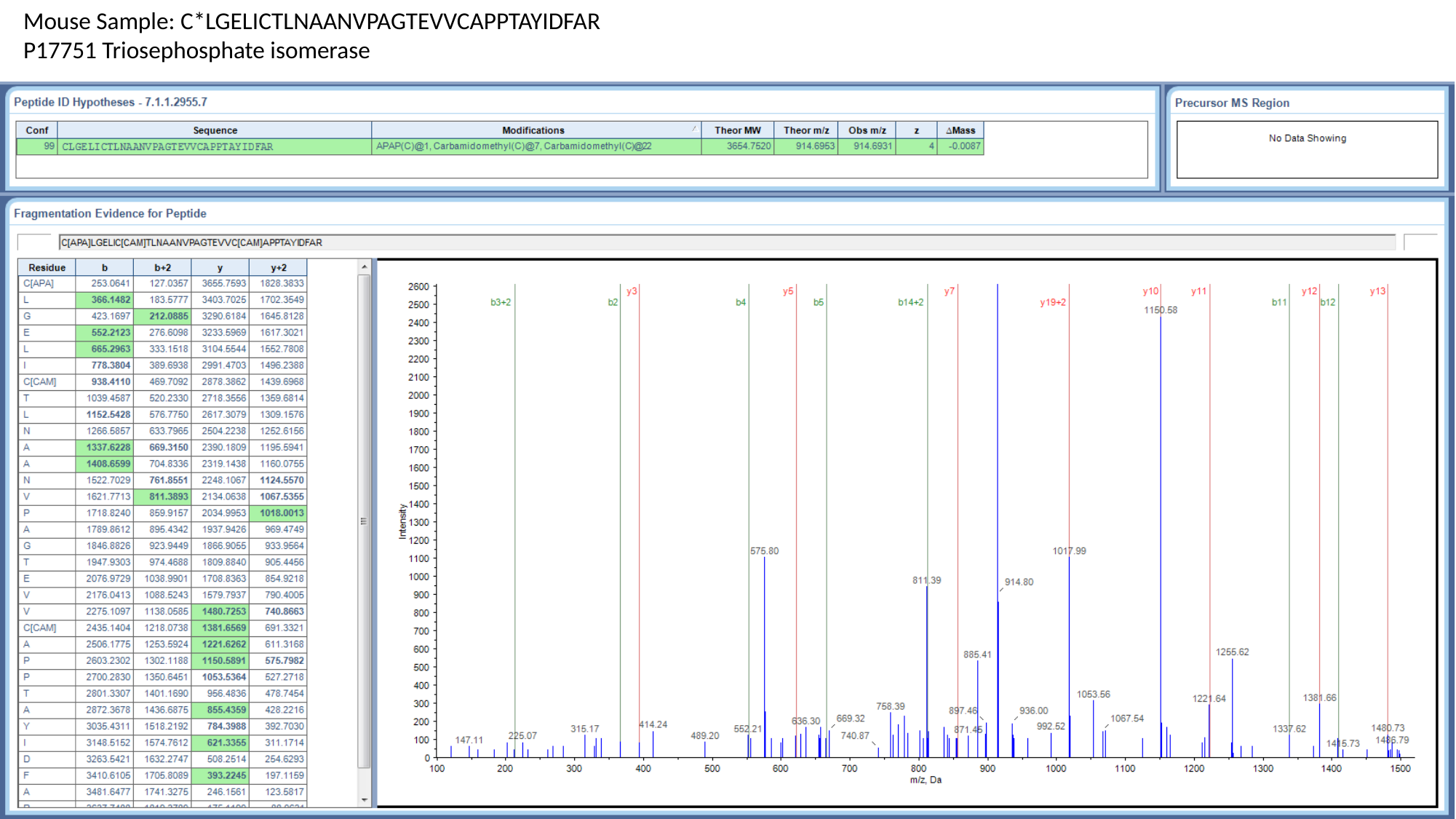

Mouse Sample: C*LGELICTLNAANVPAGTEVVCAPPTAYIDFAR
P17751 Triosephosphate isomerase

## Slide 6
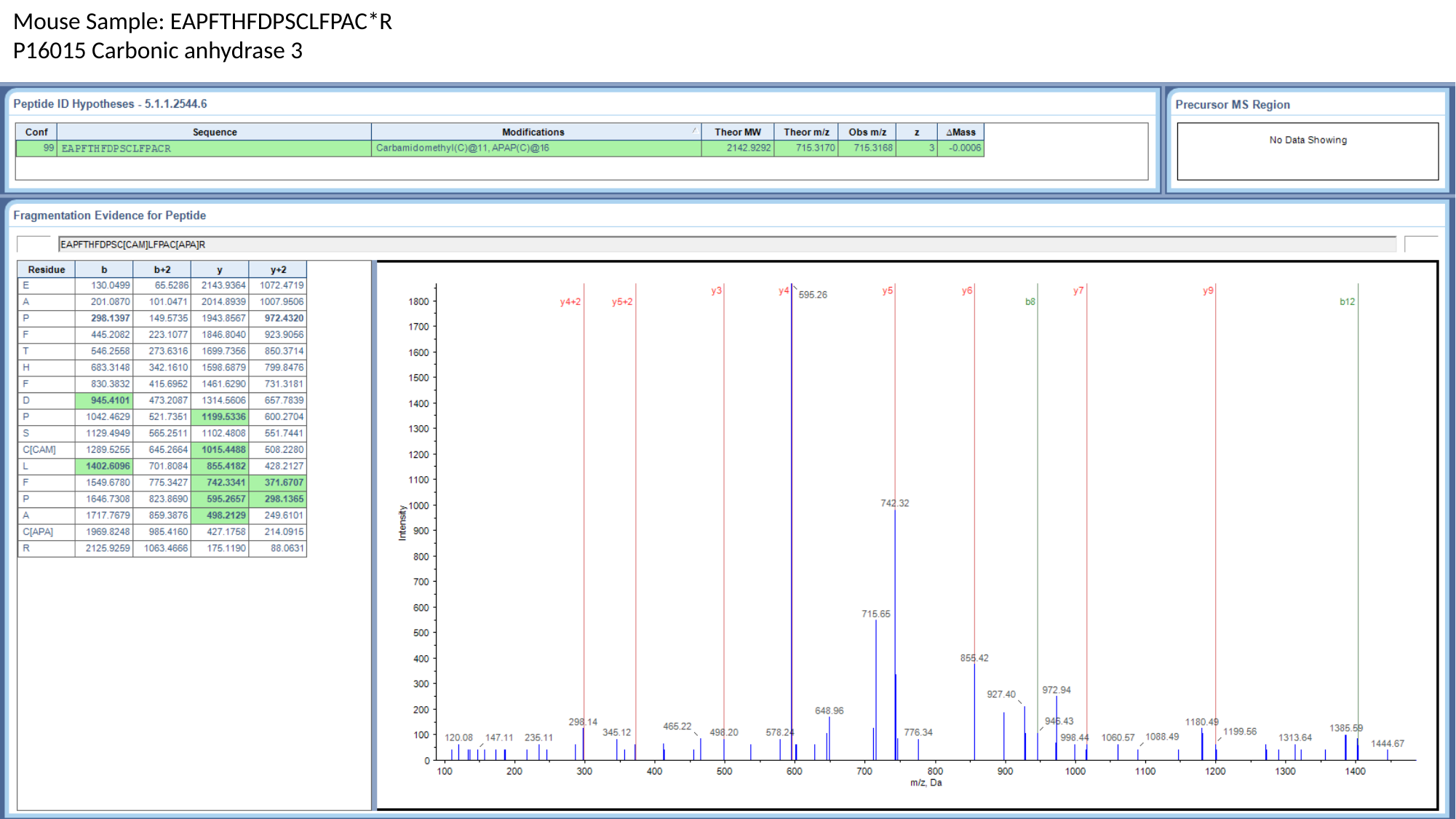

Mouse Sample: EAPFTHFDPSCLFPAC*R
P16015 Carbonic anhydrase 3

## Slide 7
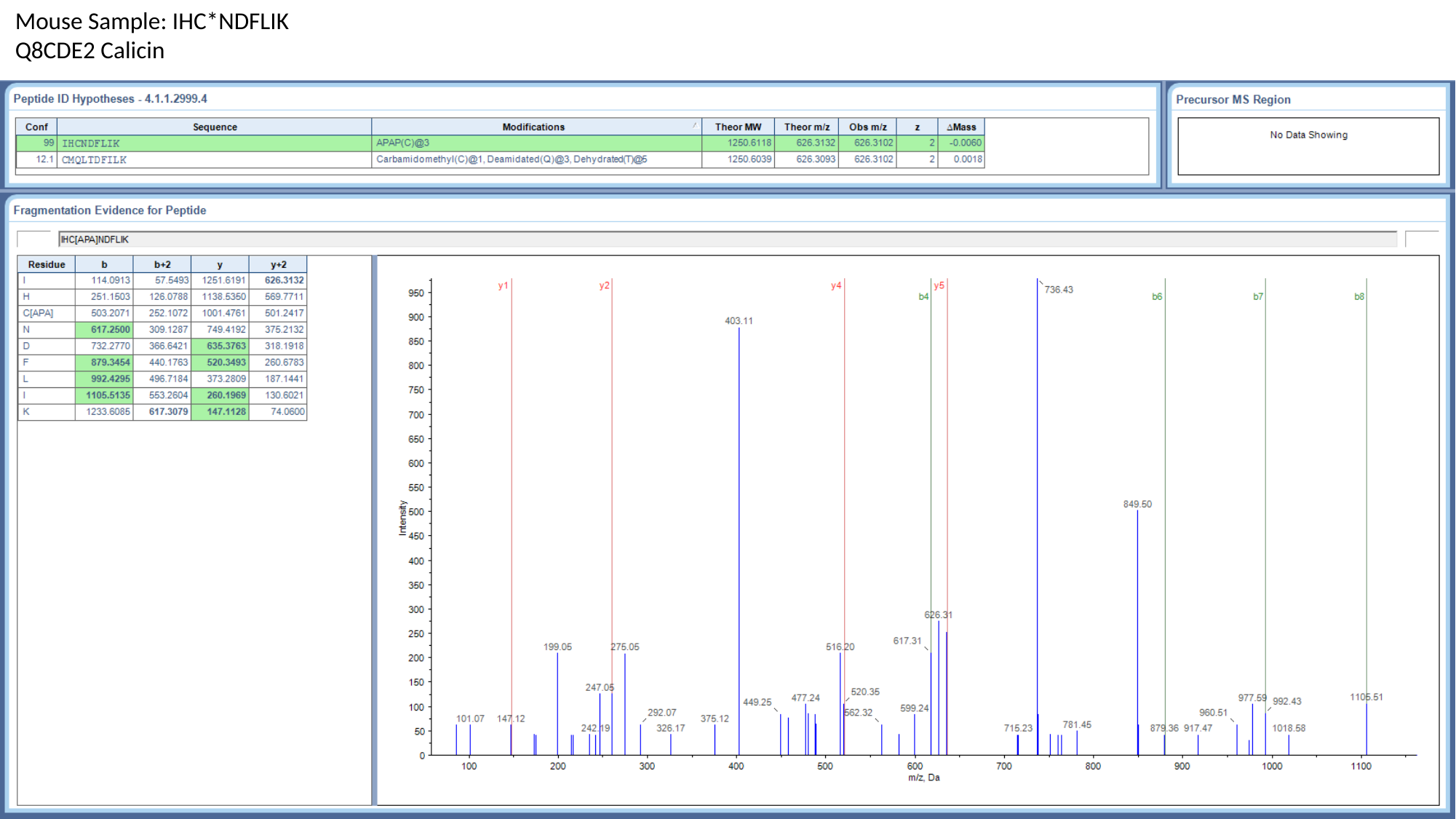

Mouse Sample: IHC*NDFLIK
Q8CDE2 Calicin

## Slide 8
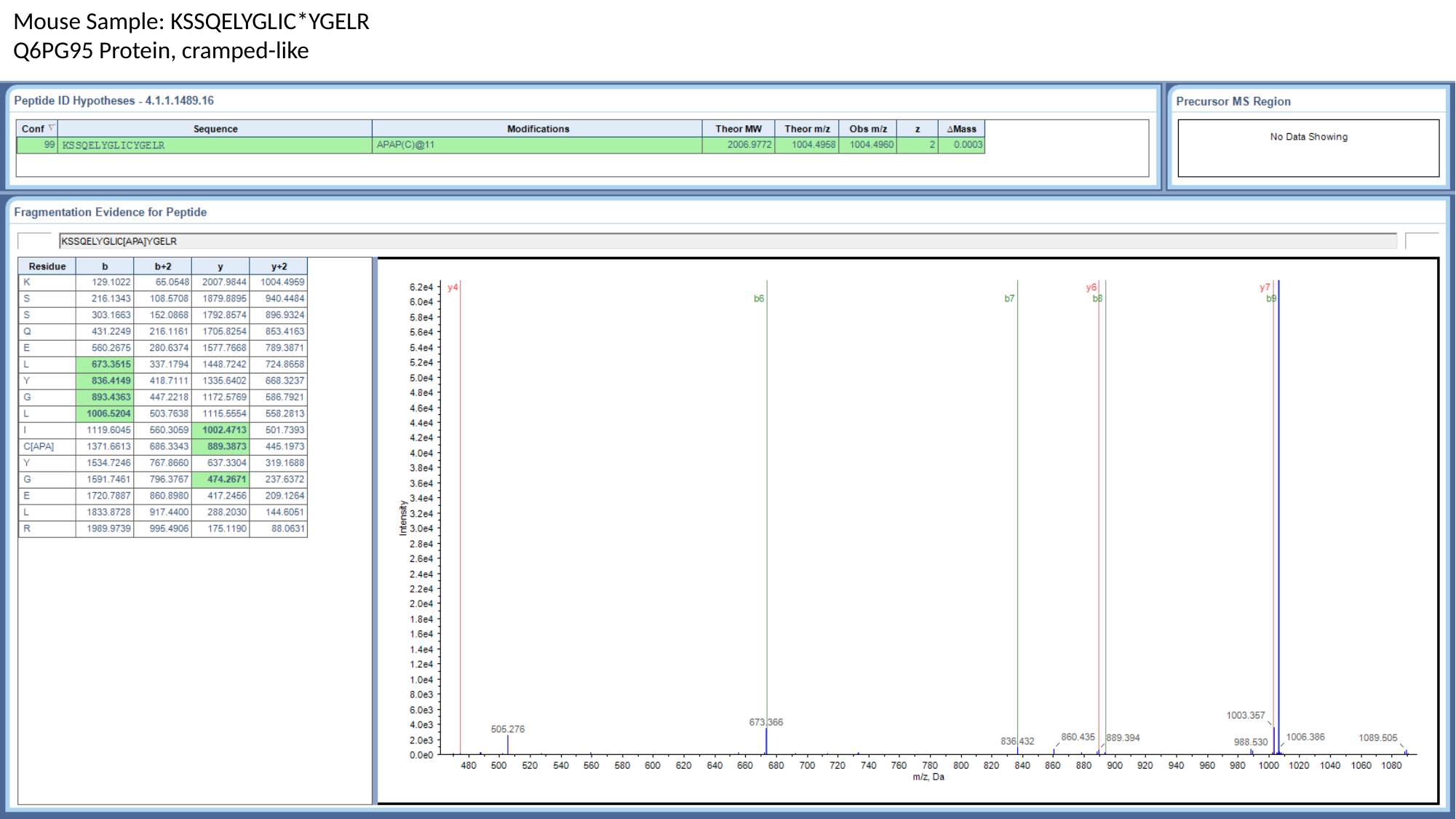

Mouse Sample: KSSQELYGLIC*YGELR
Q6PG95 Protein, cramped-like

## Slide 9
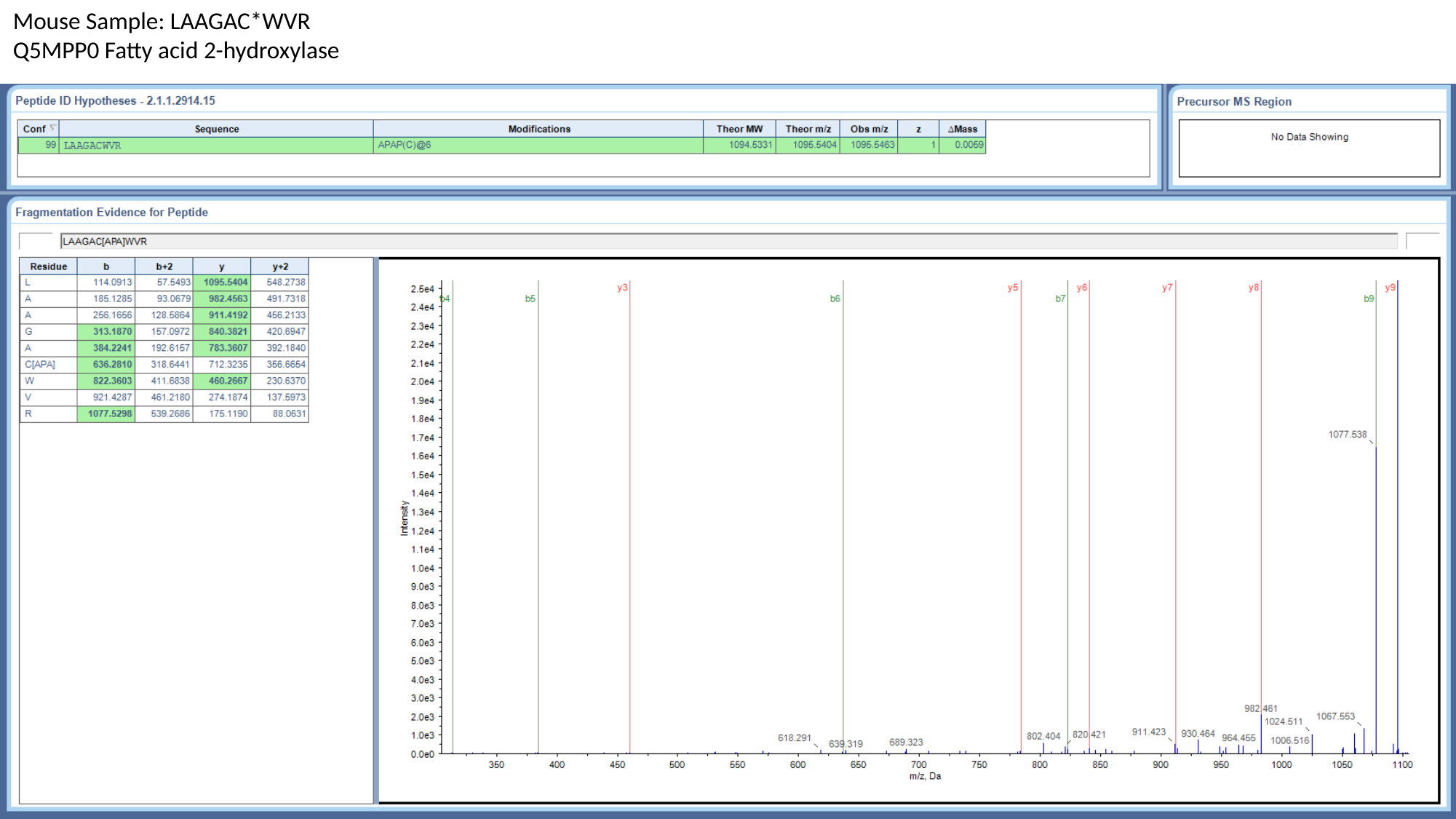

Mouse Sample: LAAGAC*WVR
Q5MPP0 Fatty acid 2-hydroxylase

## Slide 10
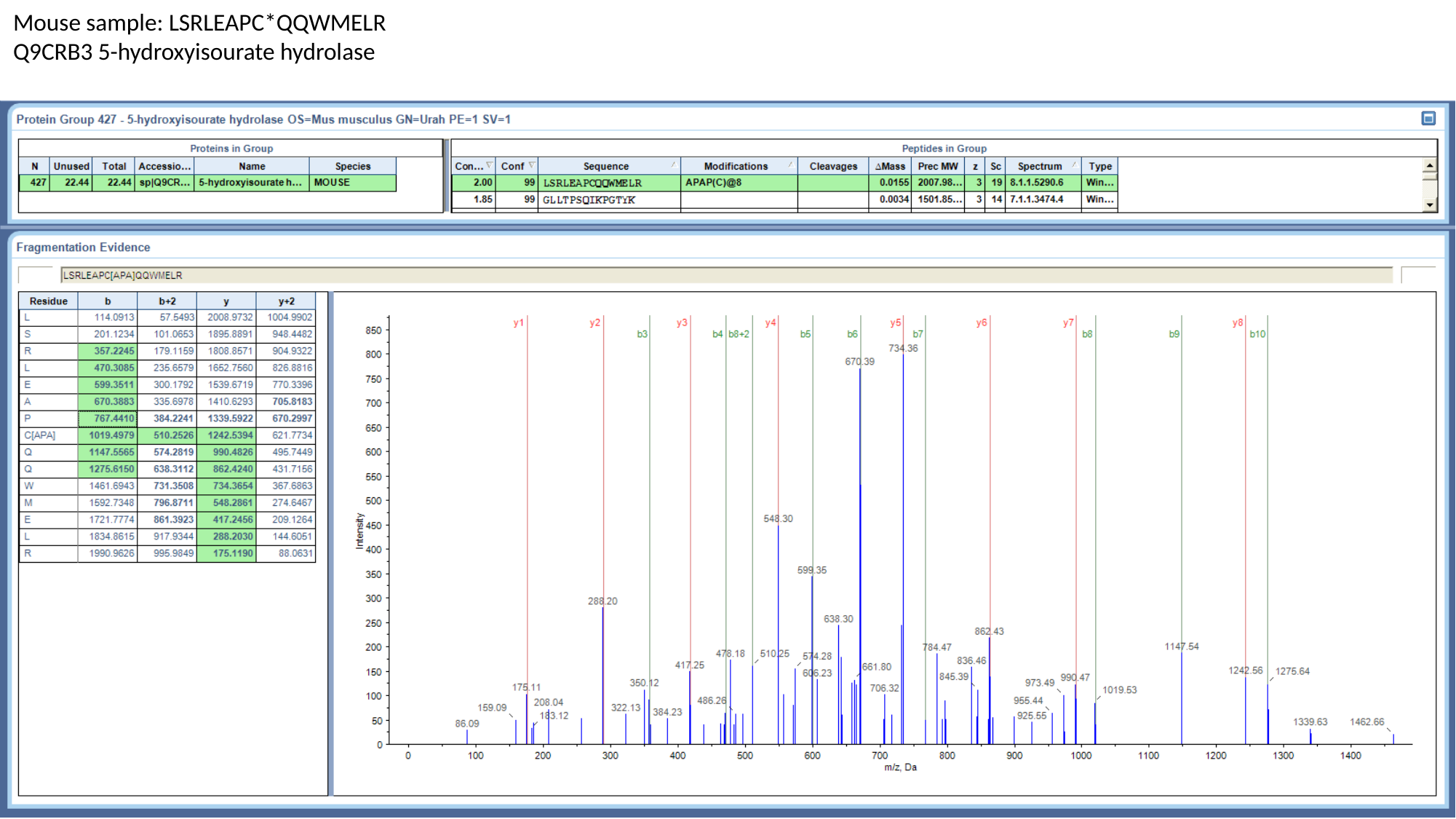

Mouse sample: LSRLEAPC*QQWMELR
Q9CRB3 5-hydroxyisourate hydrolase

## Slide 11
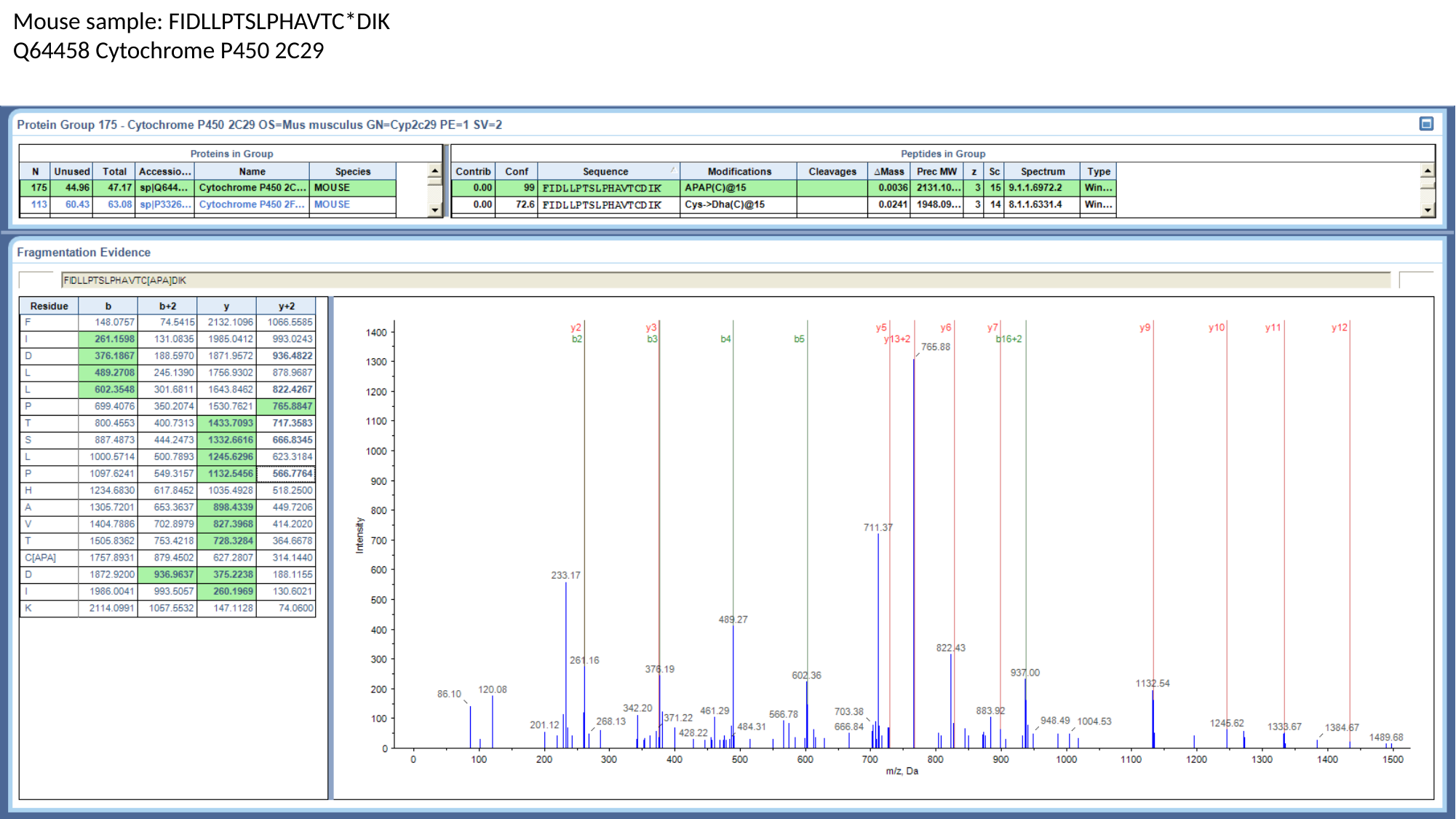

Mouse sample: FIDLLPTSLPHAVTC*DIK
Q64458 Cytochrome P450 2C29

## Slide 12
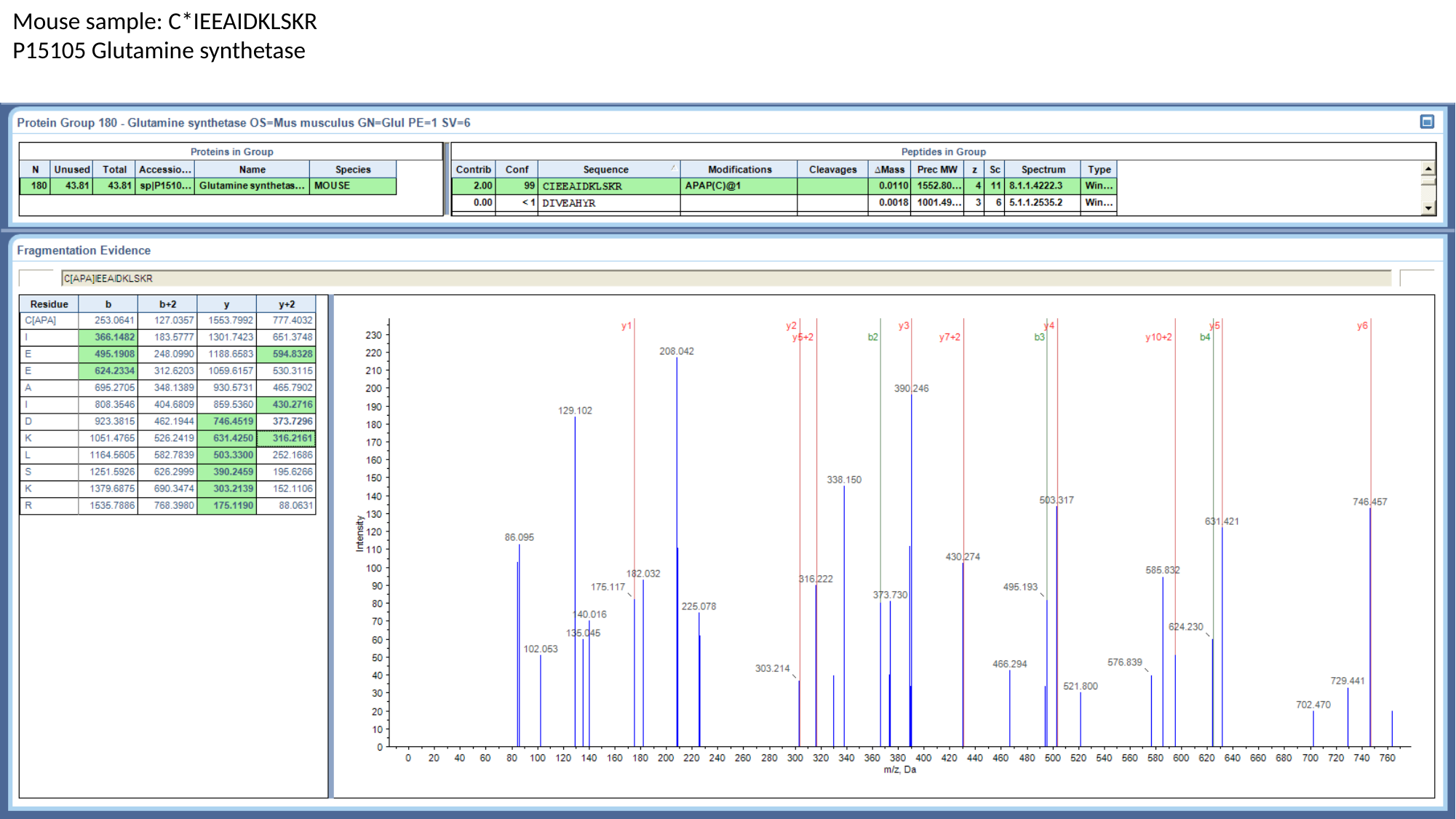

Mouse sample: C*IEEAIDKLSKR
P15105 Glutamine synthetase

## Slide 13
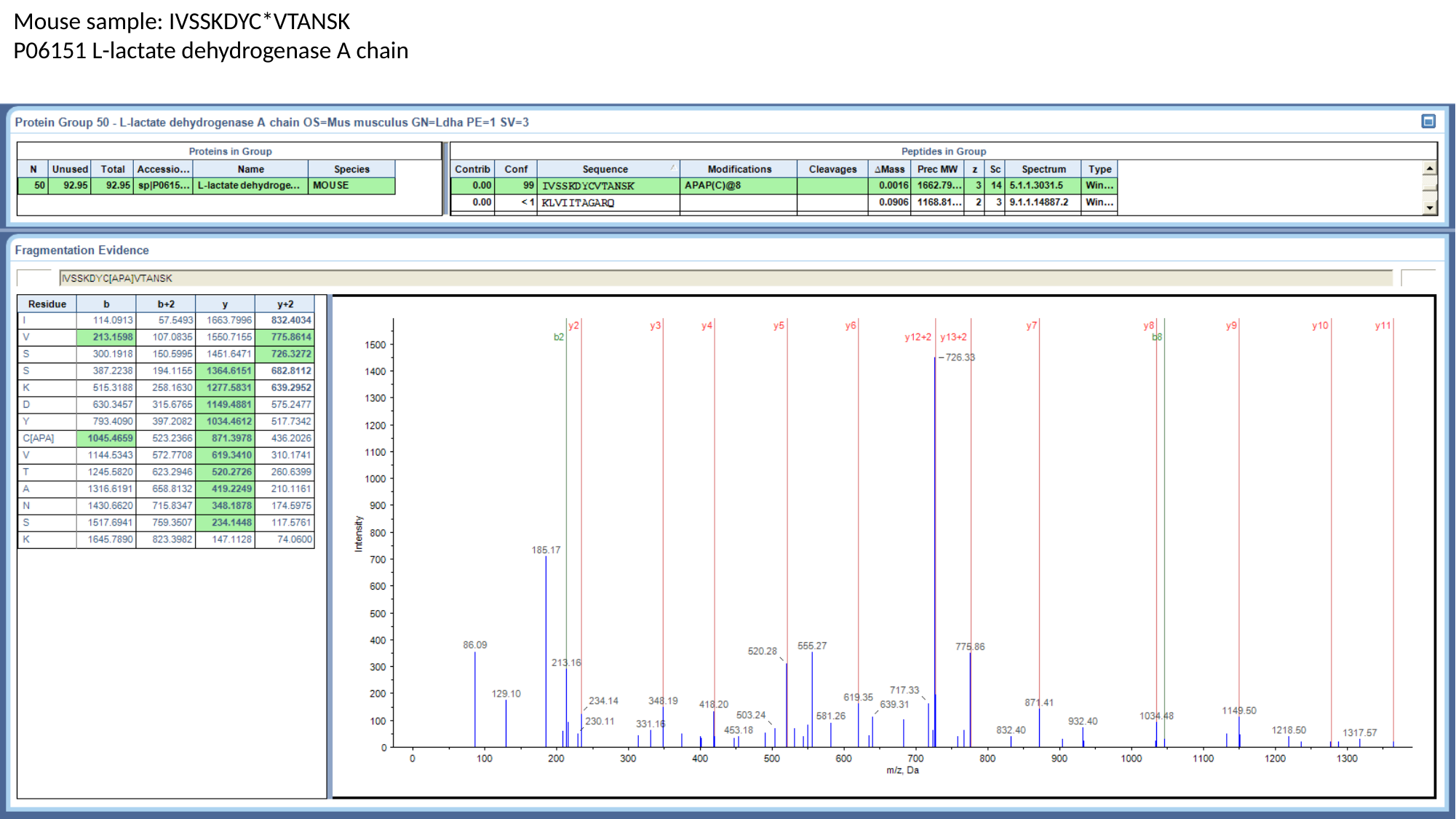

Mouse sample: IVSSKDYC*VTANSK
P06151 L-lactate dehydrogenase A chain

## Slide 14
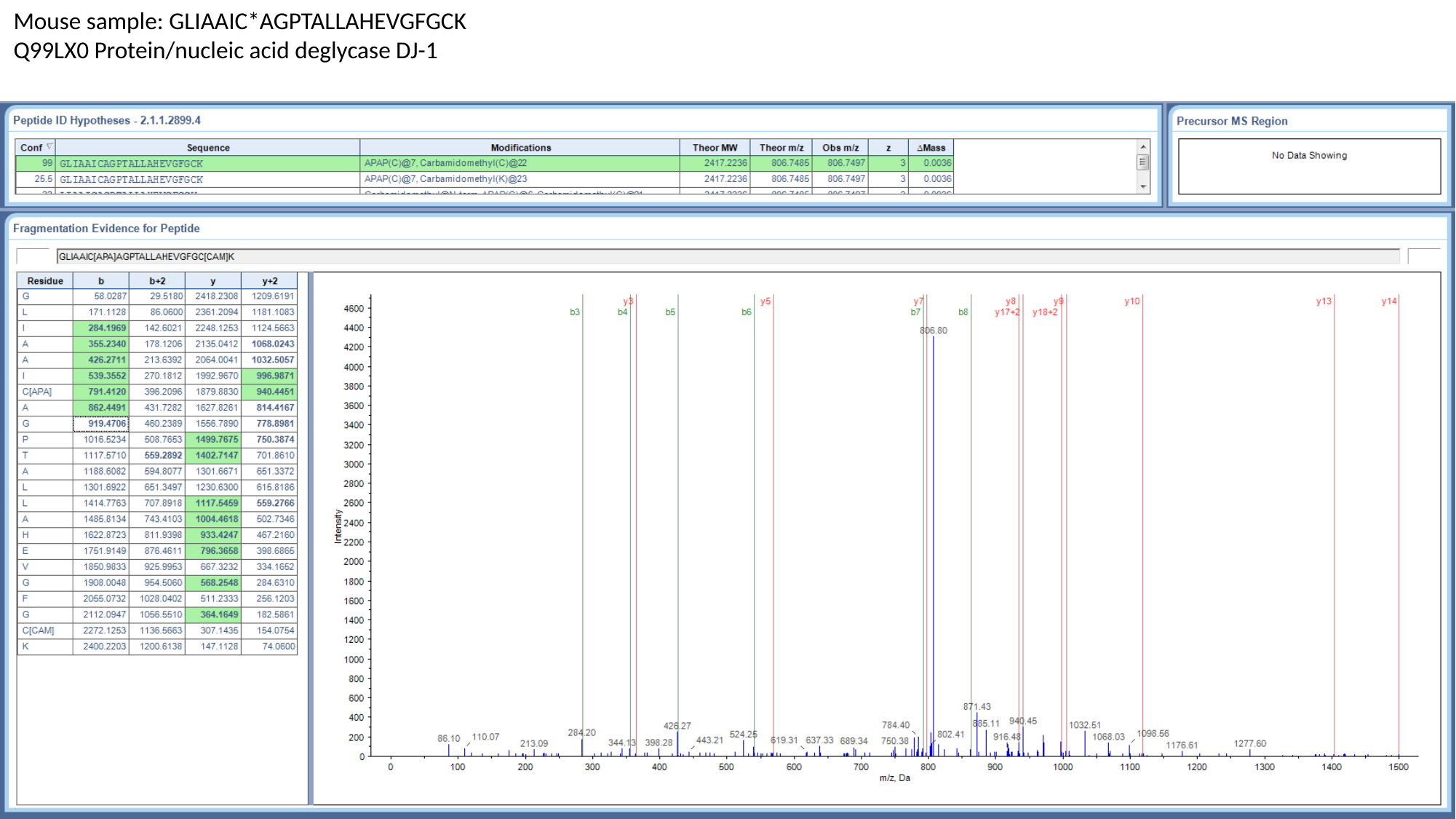

Mouse sample: GLIAAIC*AGPTALLAHEVGFGCK
Q99LX0 Protein/nucleic acid deglycase DJ-1

## Slide 15
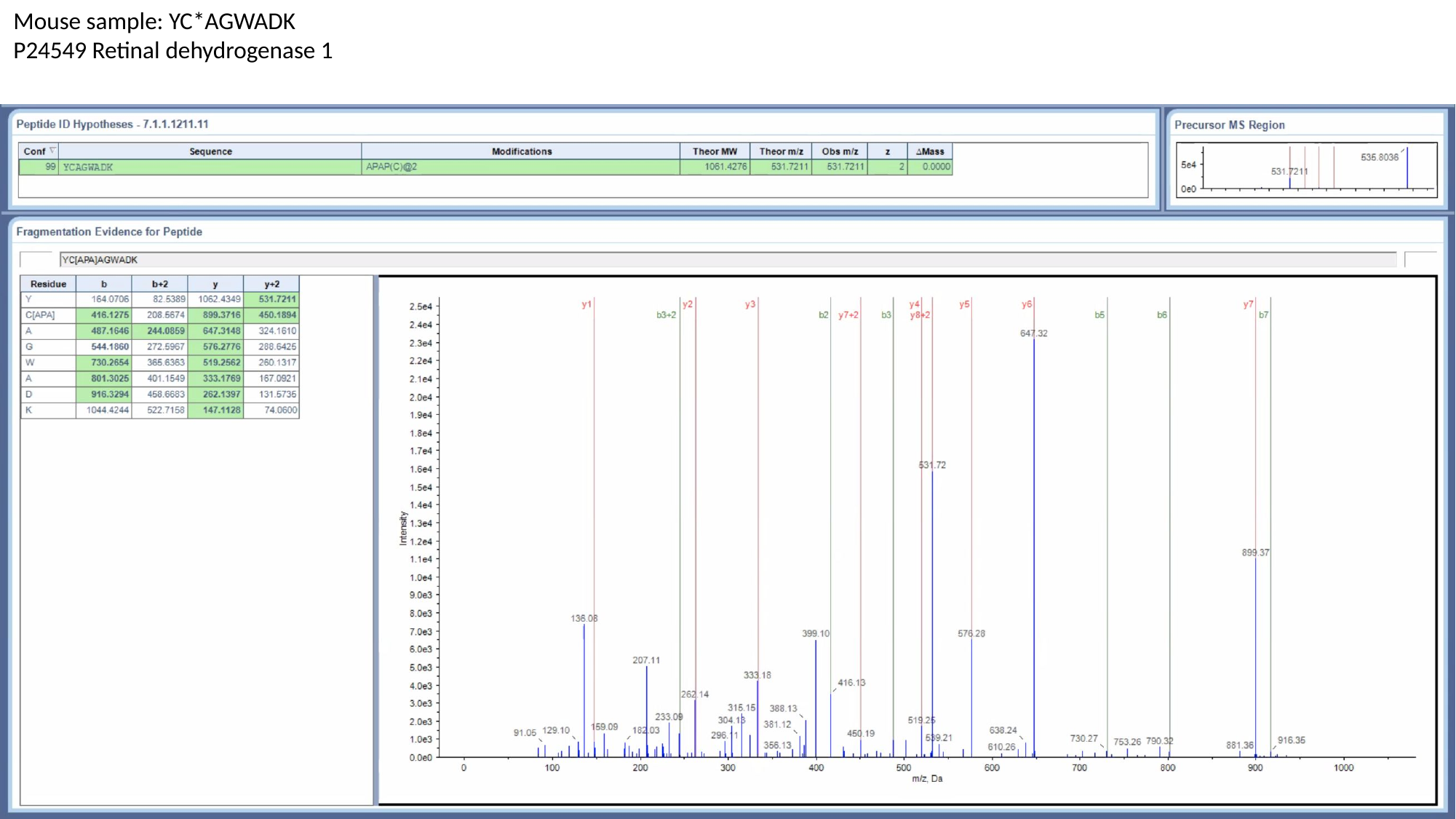

Mouse sample: YC*AGWADK
P24549 Retinal dehydrogenase 1

## Slide 16
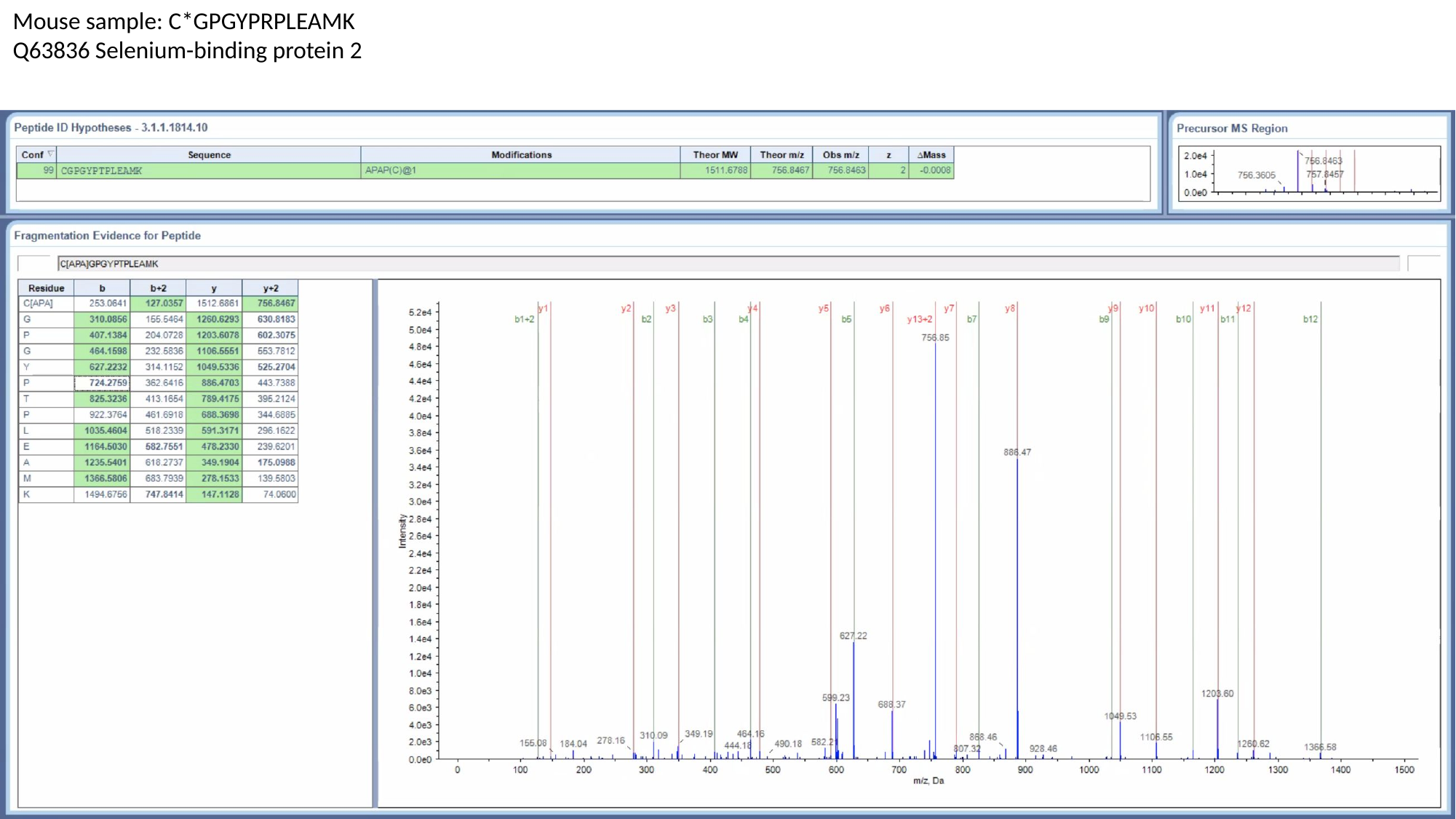

Mouse sample: C*GPGYPRPLEAMK
Q63836 Selenium-binding protein 2

## Slide 17
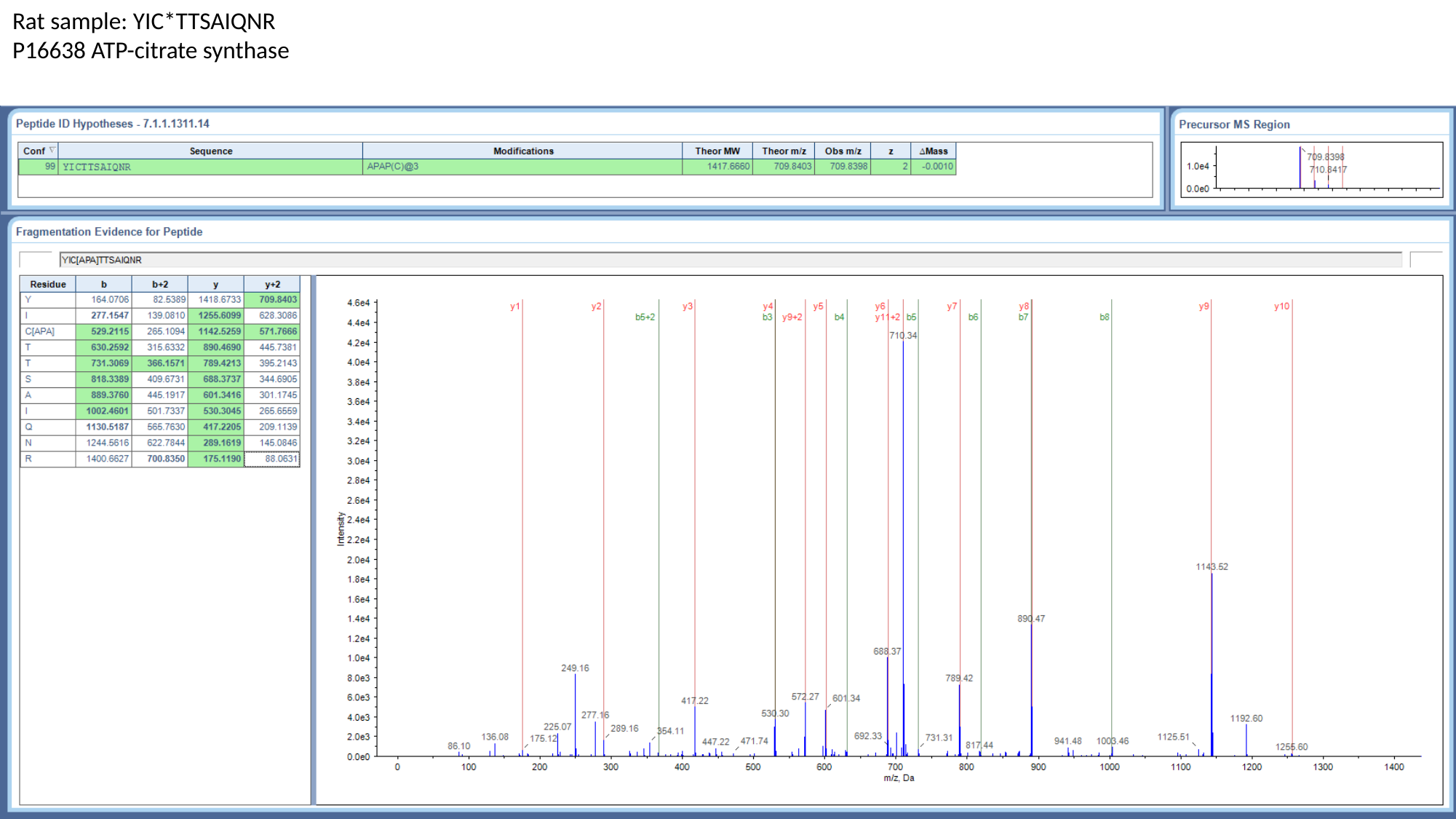

Rat sample: YIC*TTSAIQNR
P16638 ATP-citrate synthase

## Slide 18
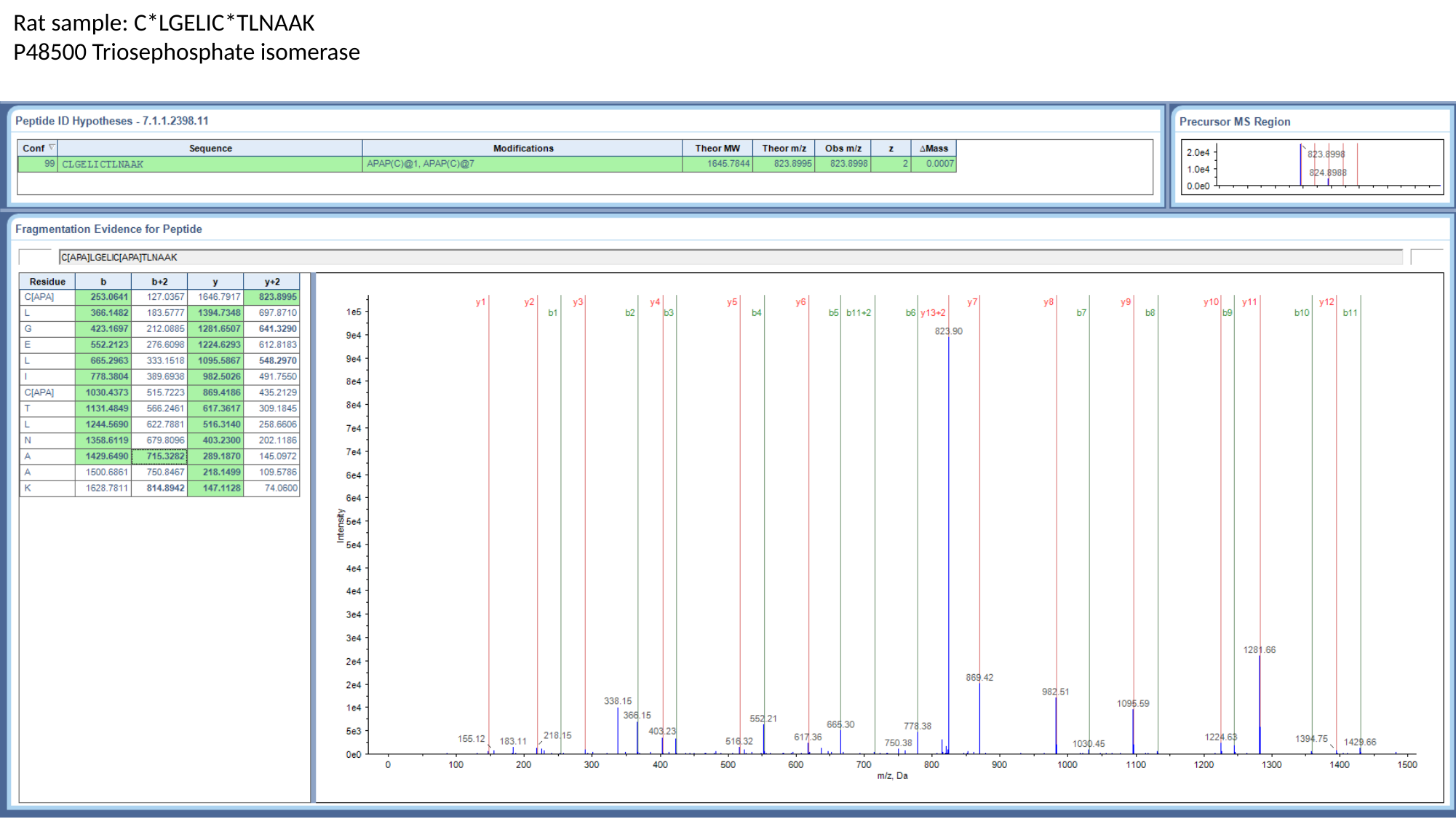

Rat sample: C*LGELIC*TLNAAK
P48500 Triosephosphate isomerase

## Slide 19
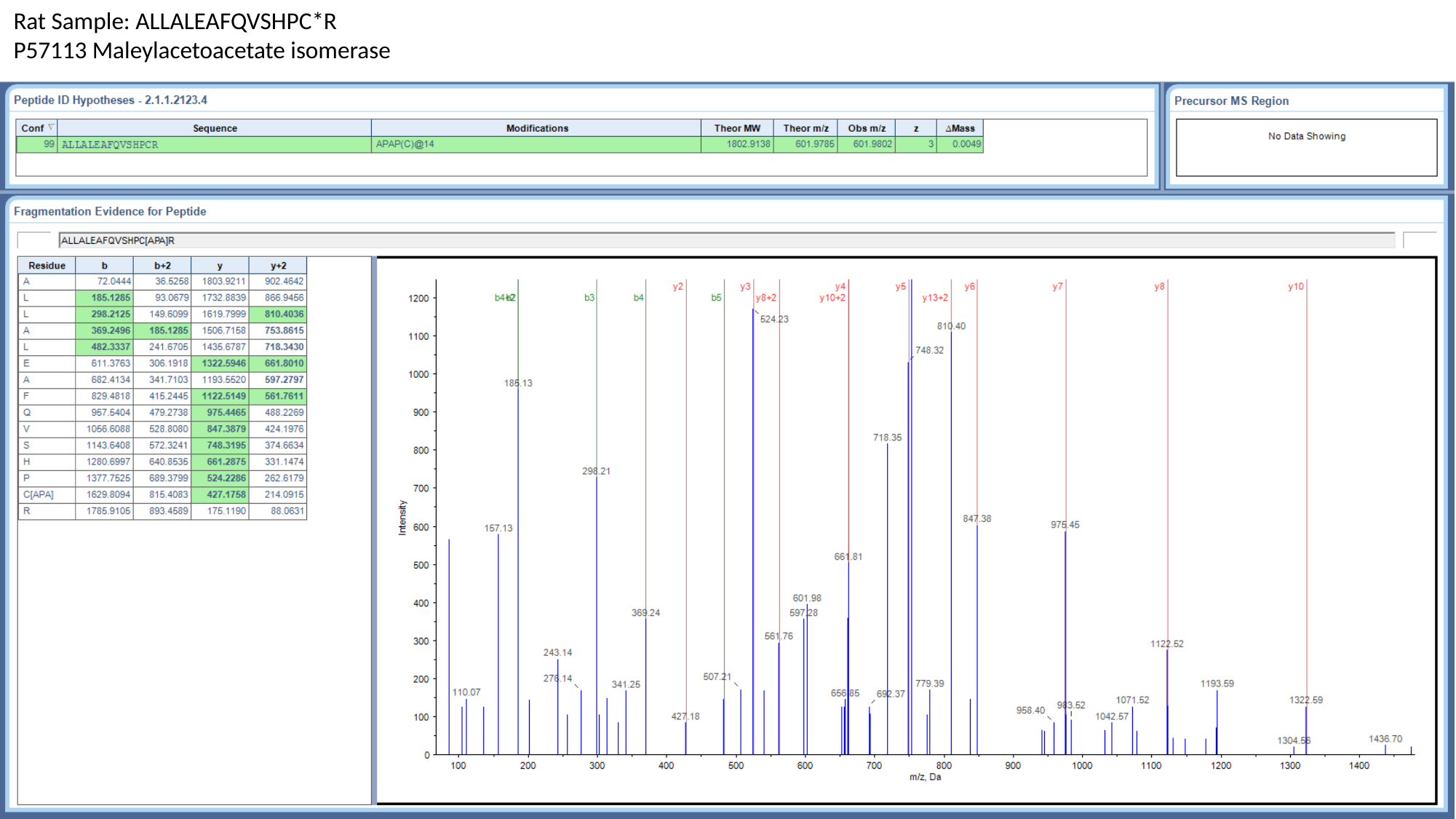

Rat Sample: ALLALEAFQVSHPC*R
P57113 Maleylacetoacetate isomerase

## Slide 20
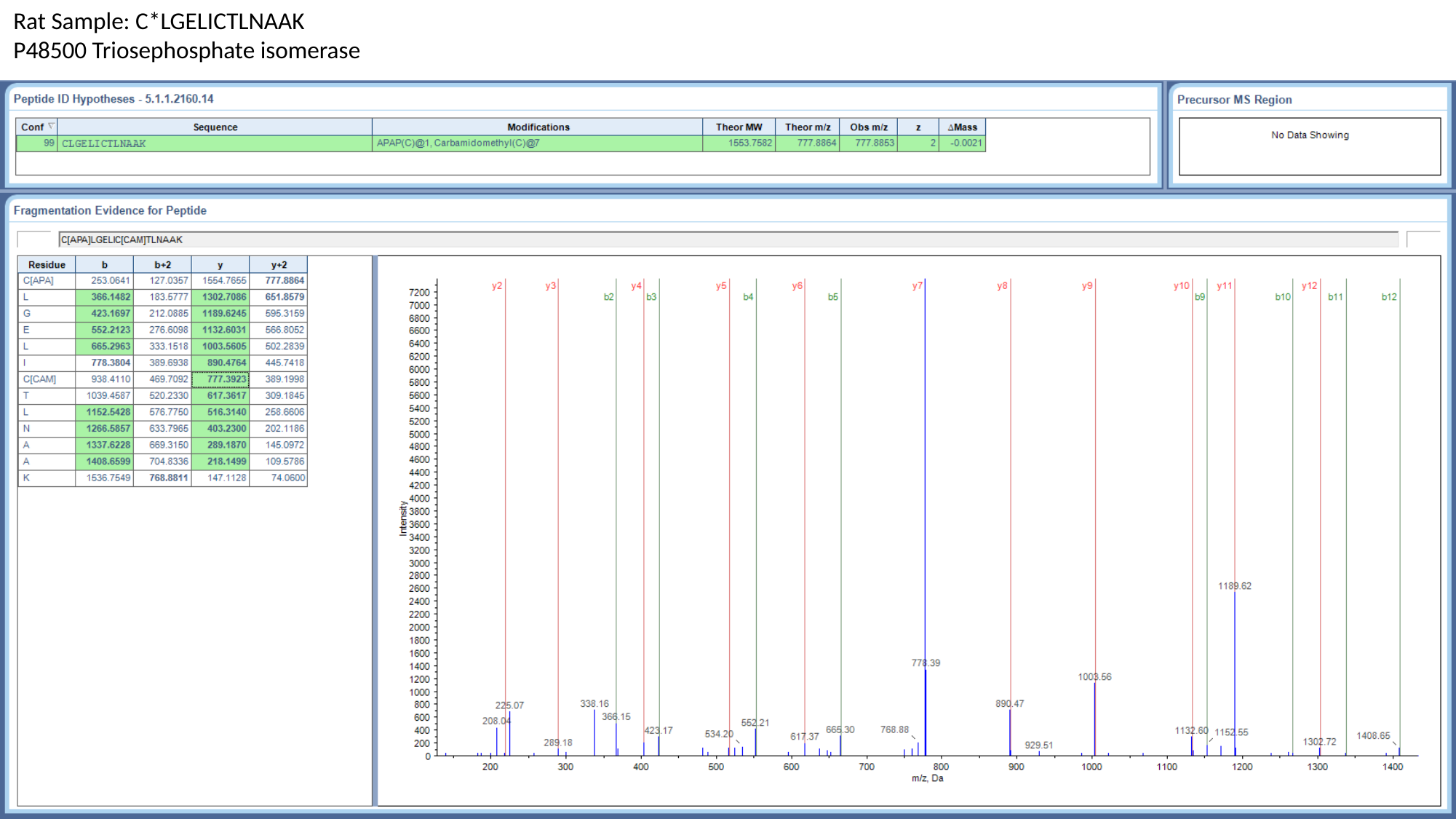

Rat Sample: C*LGELICTLNAAK
P48500 Triosephosphate isomerase

## Slide 21
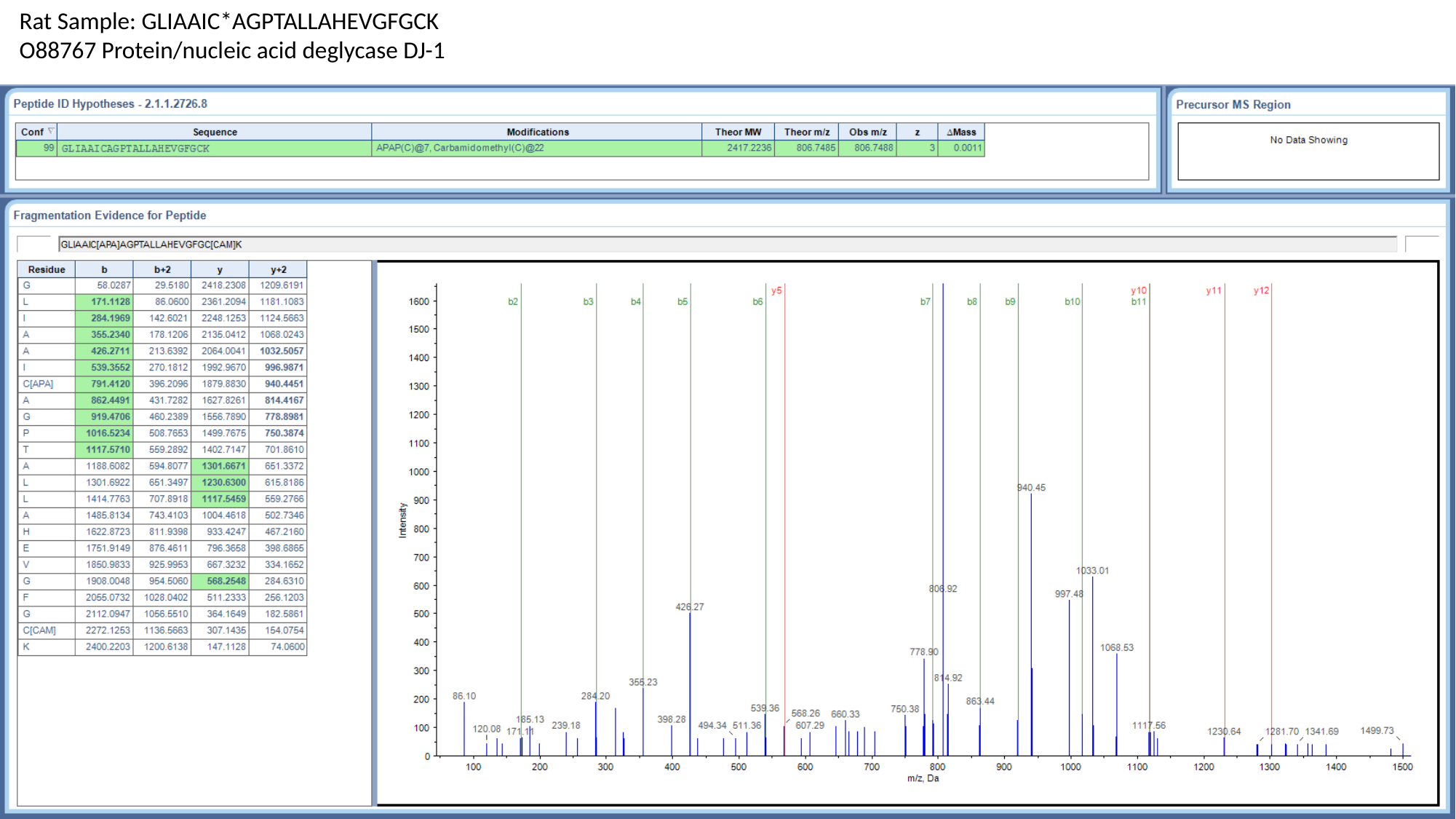

Rat Sample: GLIAAIC*AGPTALLAHEVGFGCK
O88767 Protein/nucleic acid deglycase DJ-1

## Slide 22
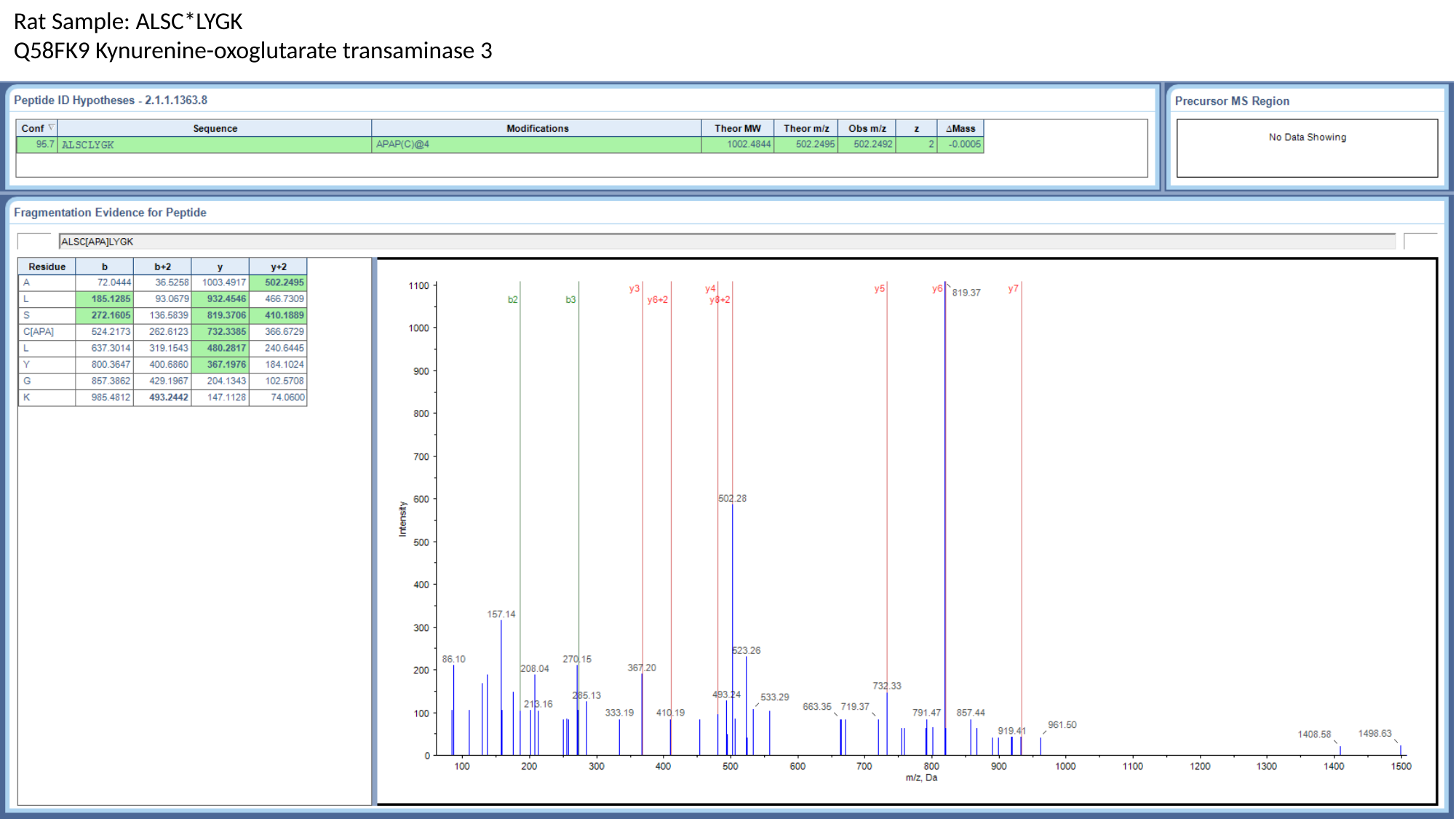

Rat Sample: ALSC*LYGK
Q58FK9 Kynurenine-oxoglutarate transaminase 3

## Slide 23
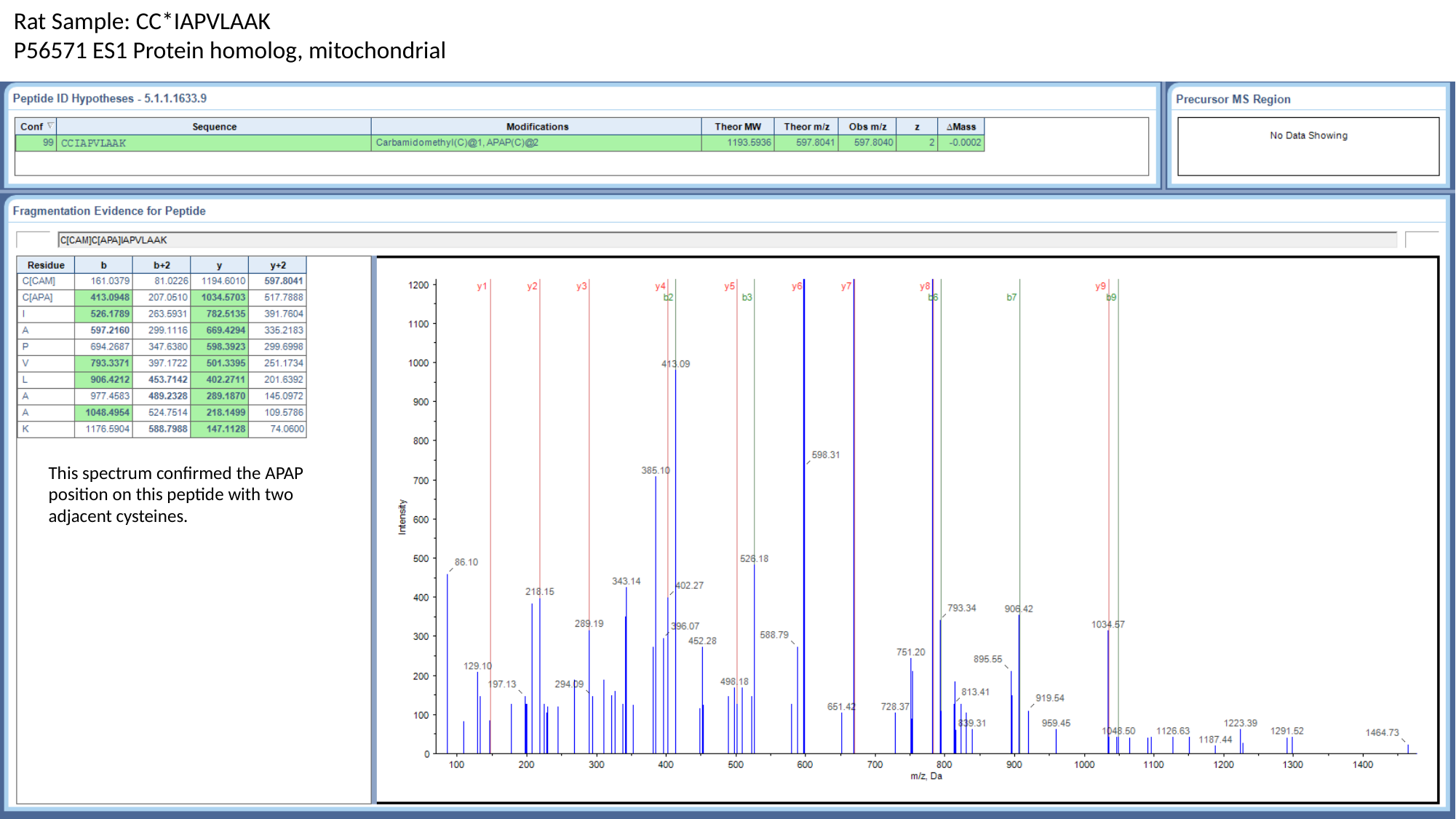

Rat Sample: CC*IAPVLAAK
P56571 ES1 Protein homolog, mitochondrial
This spectrum confirmed the APAP position on this peptide with two adjacent cysteines.

## Slide 24
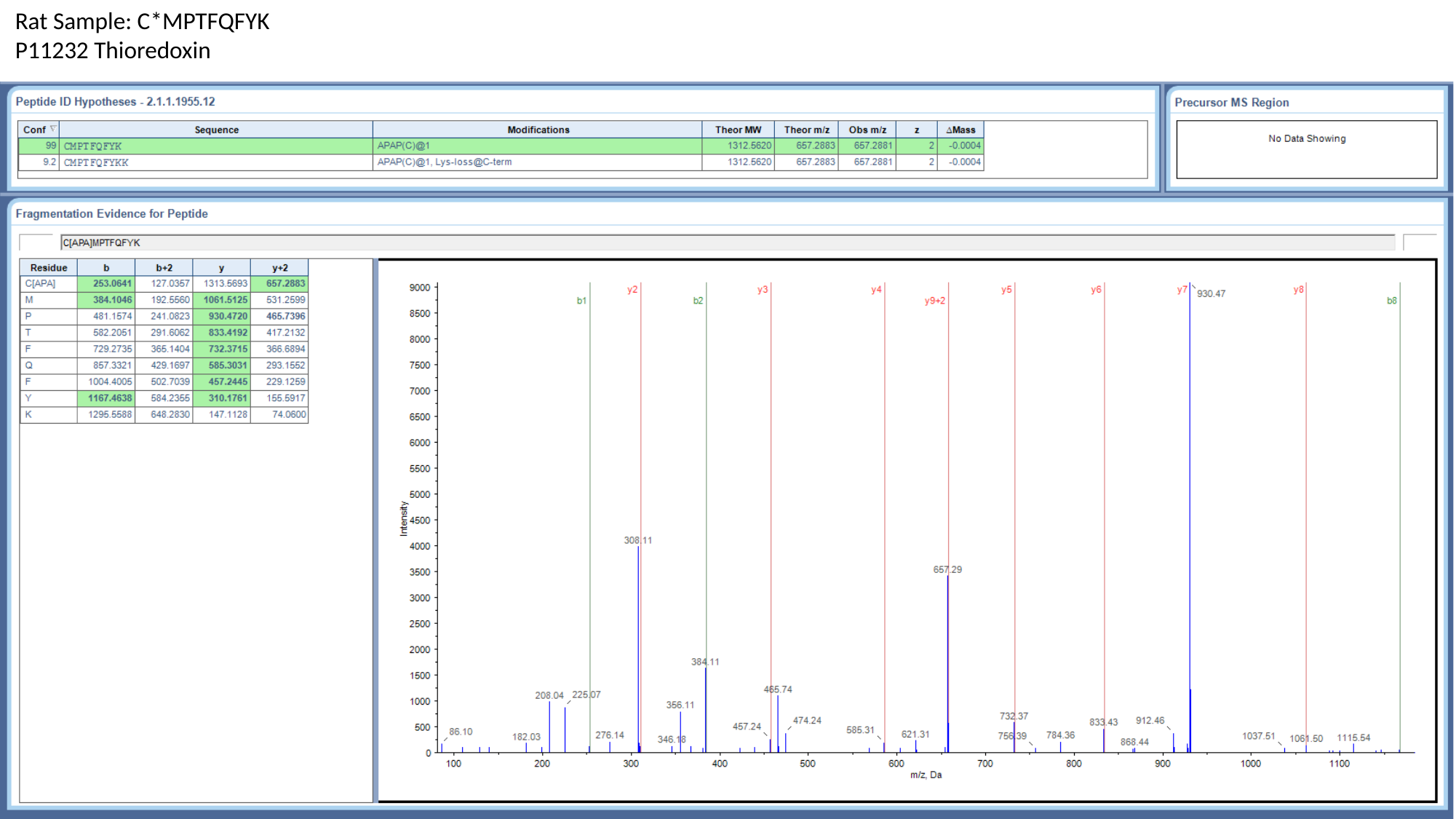

Rat Sample: C*MPTFQFYK
P11232 Thioredoxin

## Slide 25
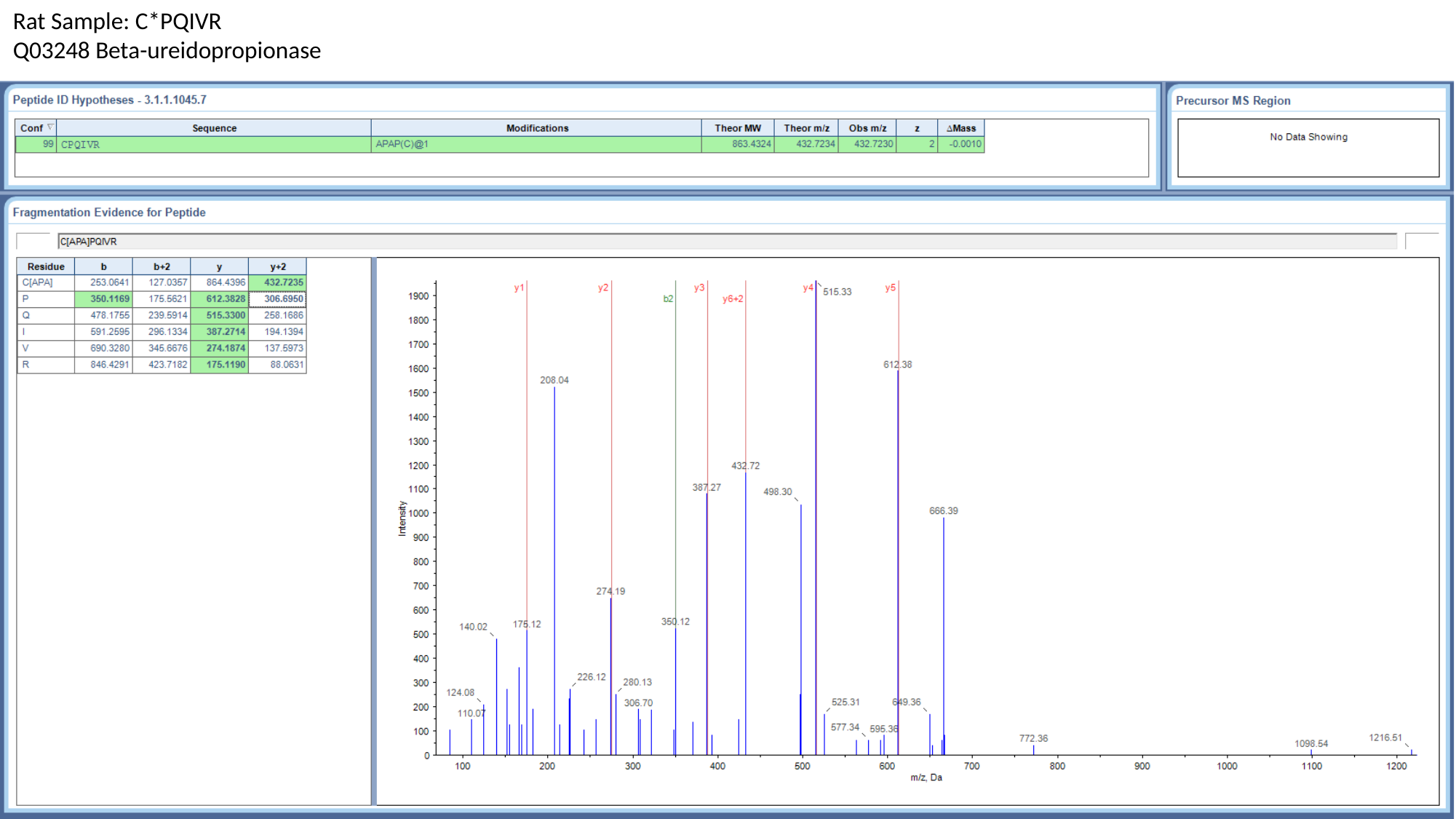

Rat Sample: C*PQIVR
Q03248 Beta-ureidopropionase

## Slide 26
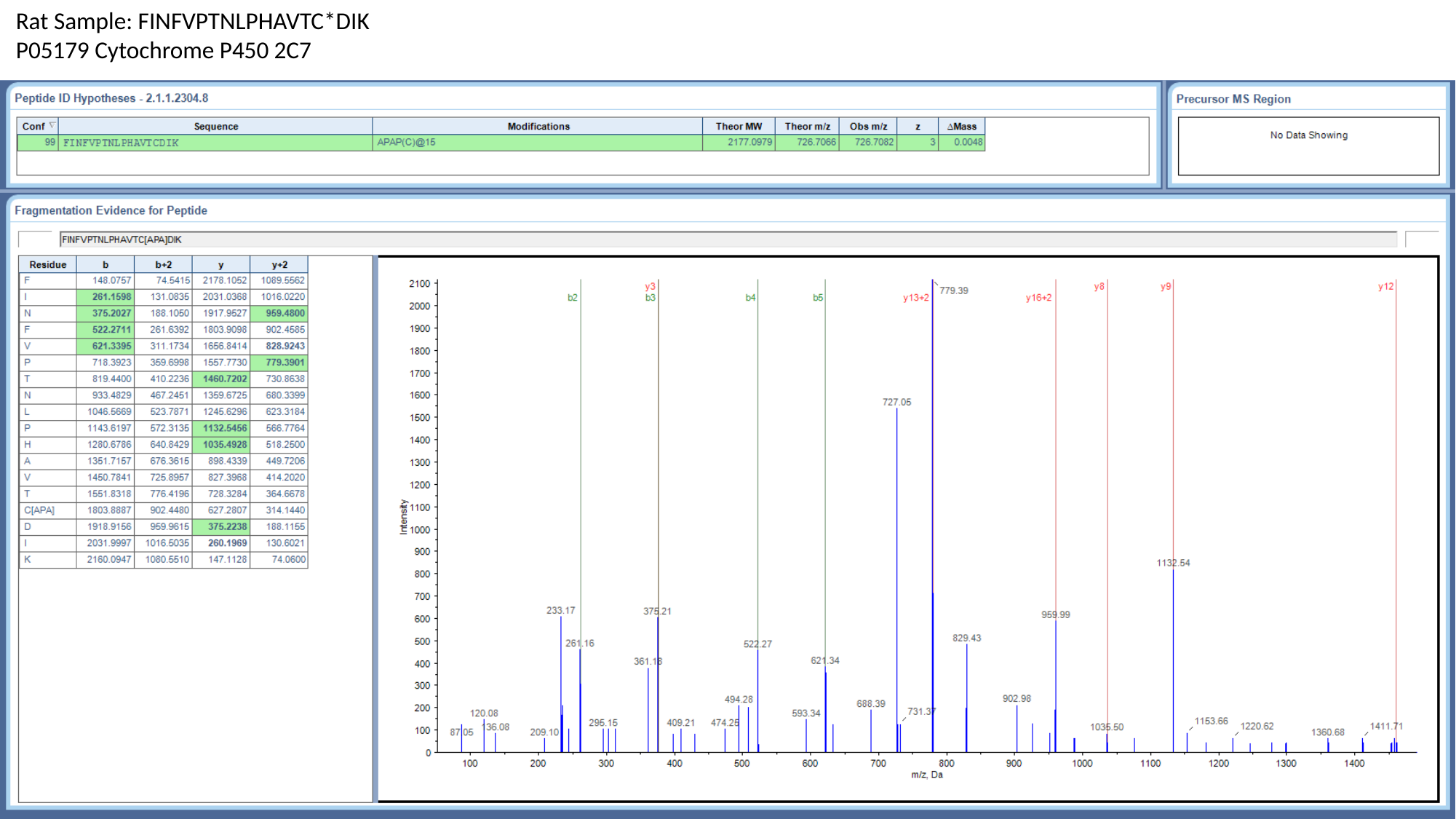

Rat Sample: FINFVPTNLPHAVTC*DIK
P05179 Cytochrome P450 2C7

## Slide 27
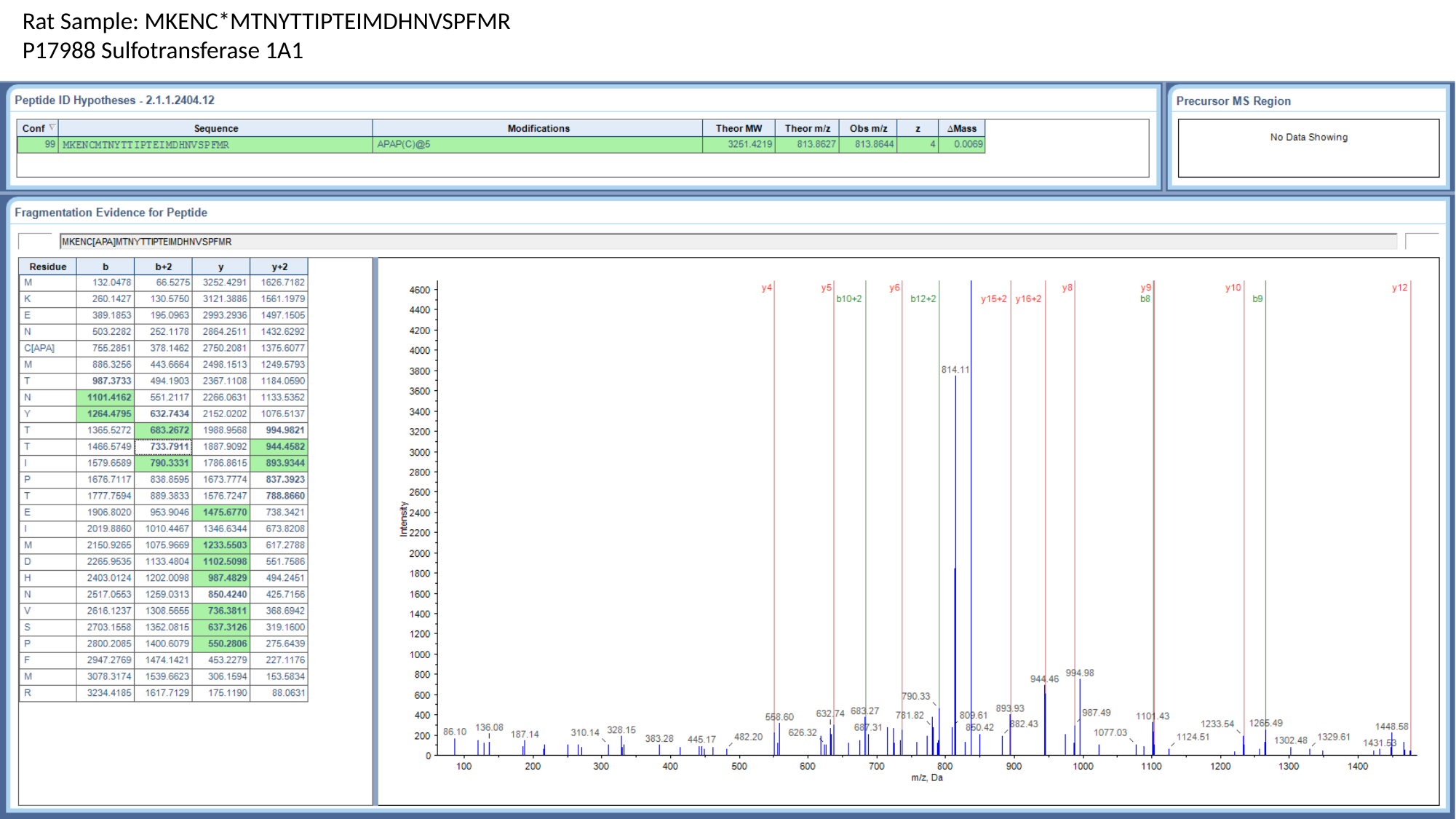

Rat Sample: MKENC*MTNYTTIPTEIMDHNVSPFMR
P17988 Sulfotransferase 1A1

## Slide 28
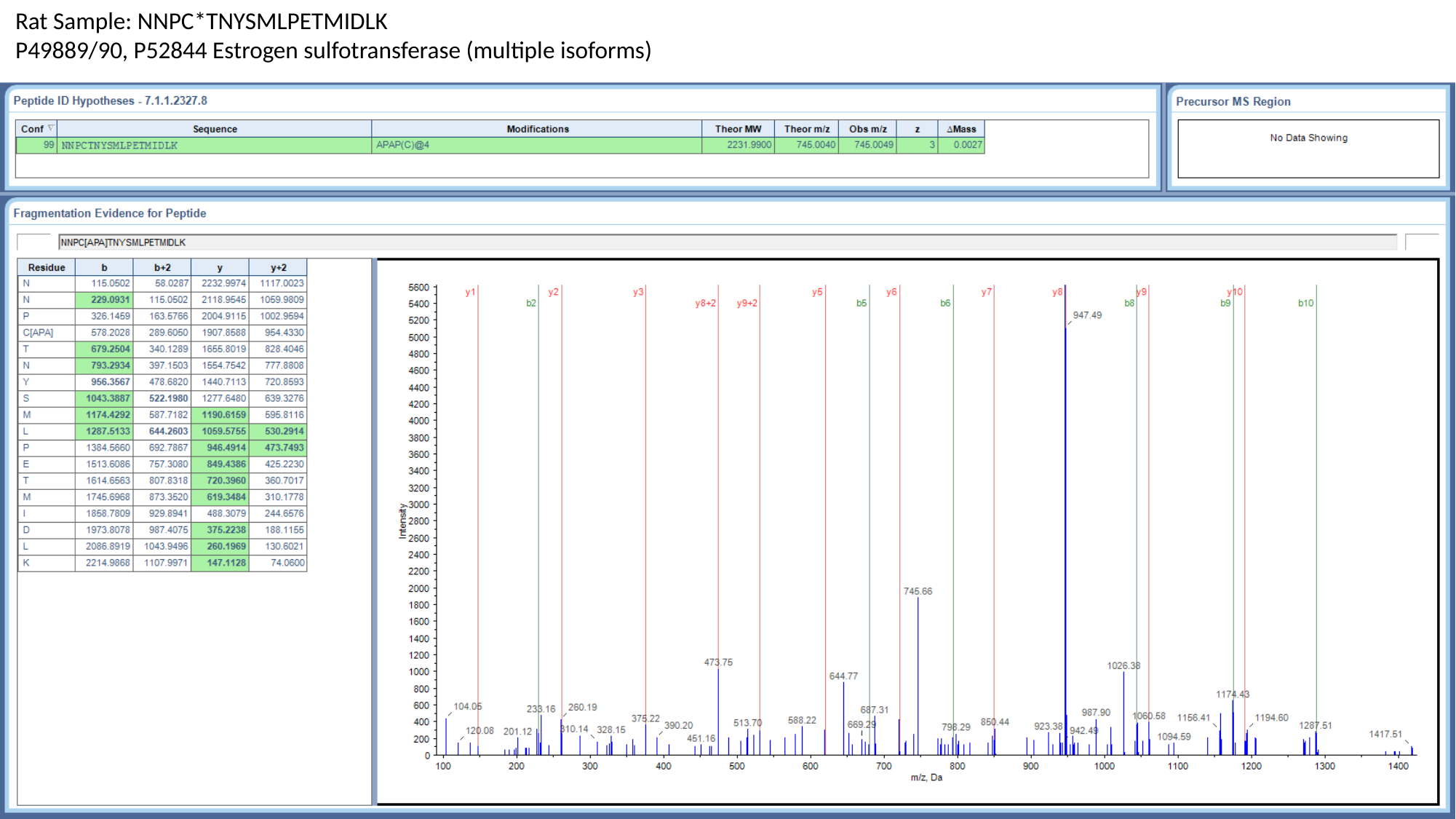

Rat Sample: NNPC*TNYSMLPETMIDLK
P49889/90, P52844 Estrogen sulfotransferase (multiple isoforms)

## Slide 29
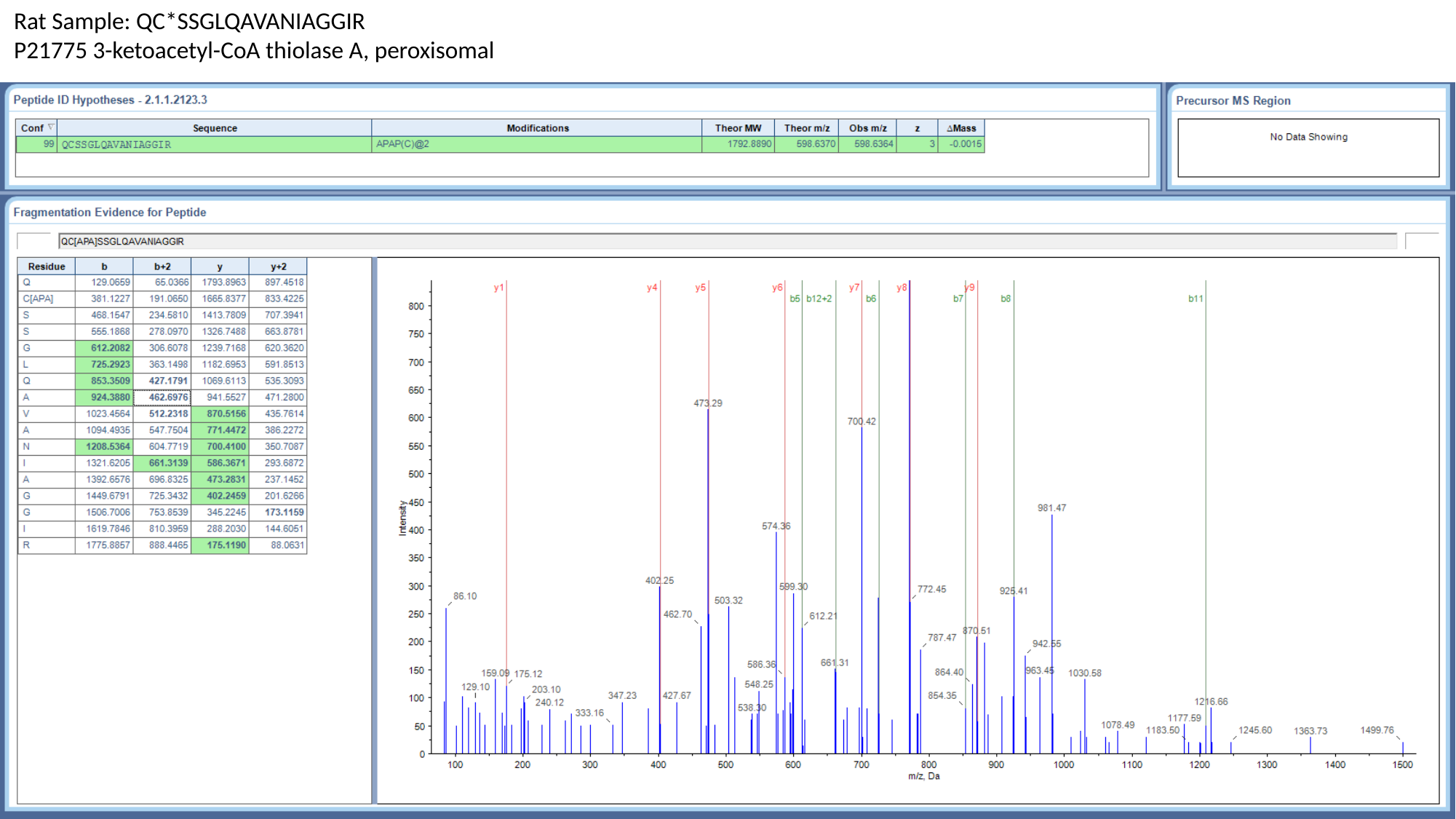

Rat Sample: QC*SSGLQAVANIAGGIR
P21775 3-ketoacetyl-CoA thiolase A, peroxisomal

## Slide 30
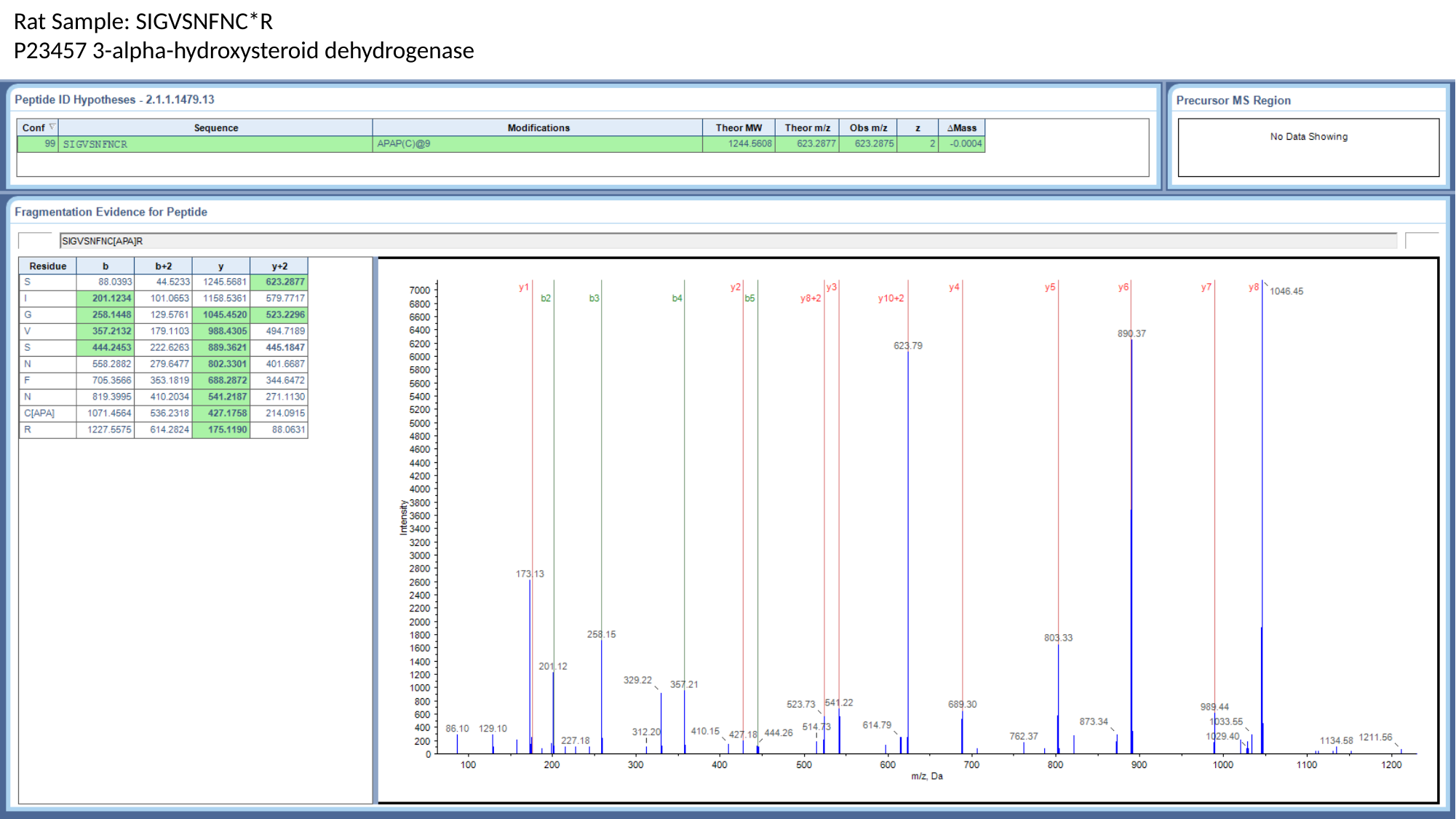

Rat Sample: SIGVSNFNC*R
P23457 3-alpha-hydroxysteroid dehydrogenase

## Slide 31
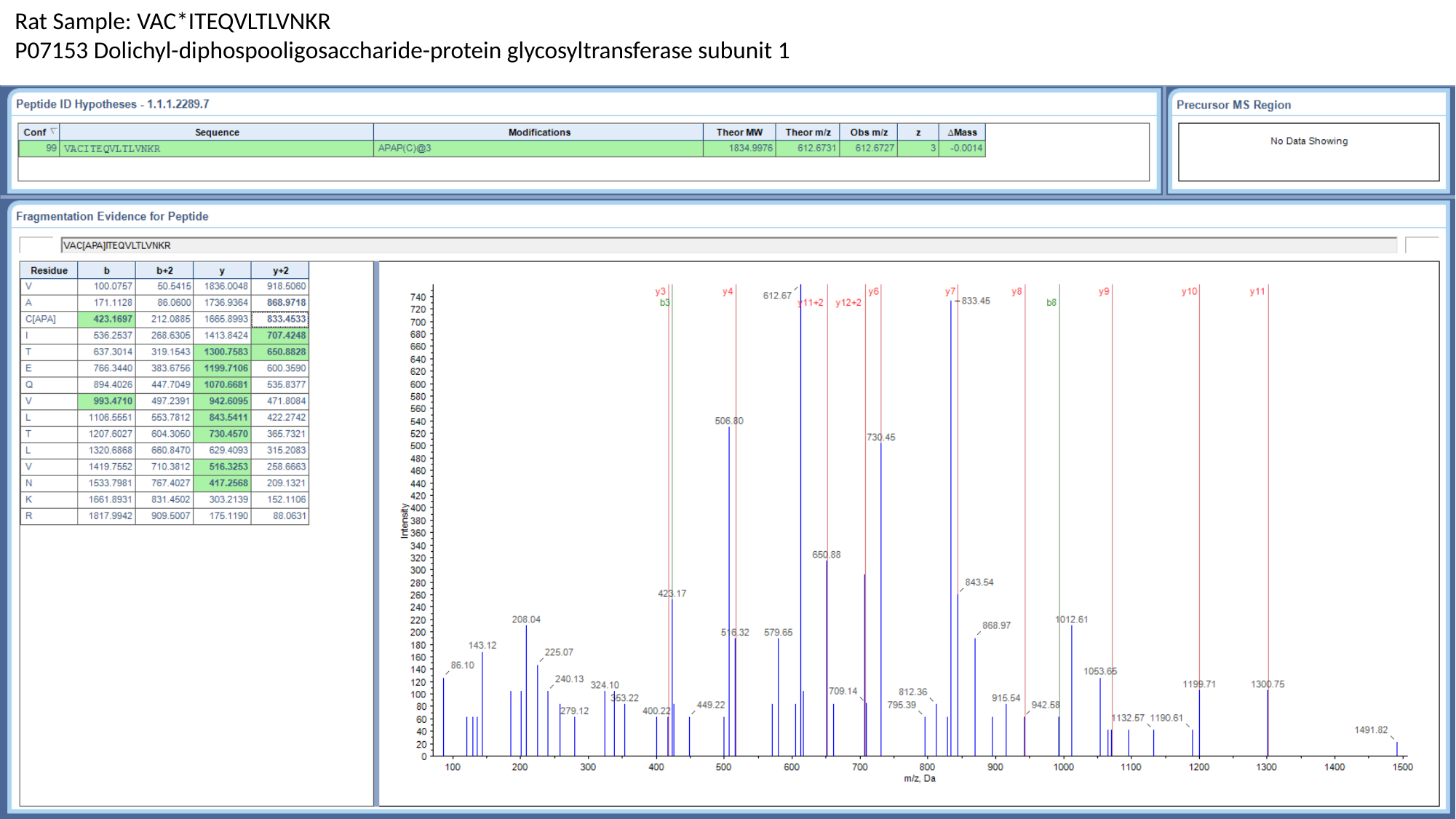

Rat Sample: VAC*ITEQVLTLVNKR
P07153 Dolichyl-diphospooligosaccharide-protein glycosyltransferase subunit 1

## Slide 32
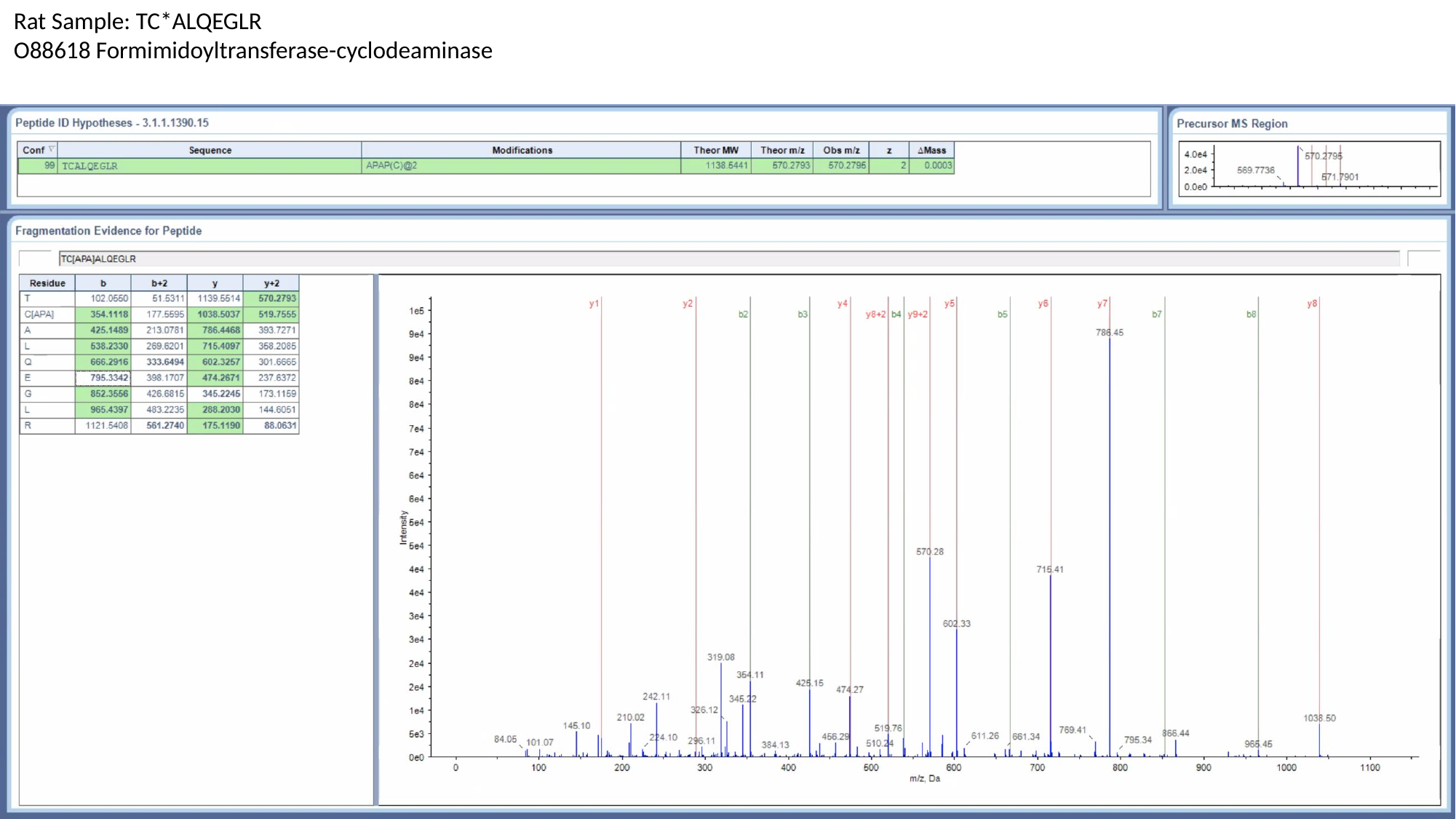

Rat Sample: TC*ALQEGLR
O88618 Formimidoyltransferase-cyclodeaminase

## Slide 33
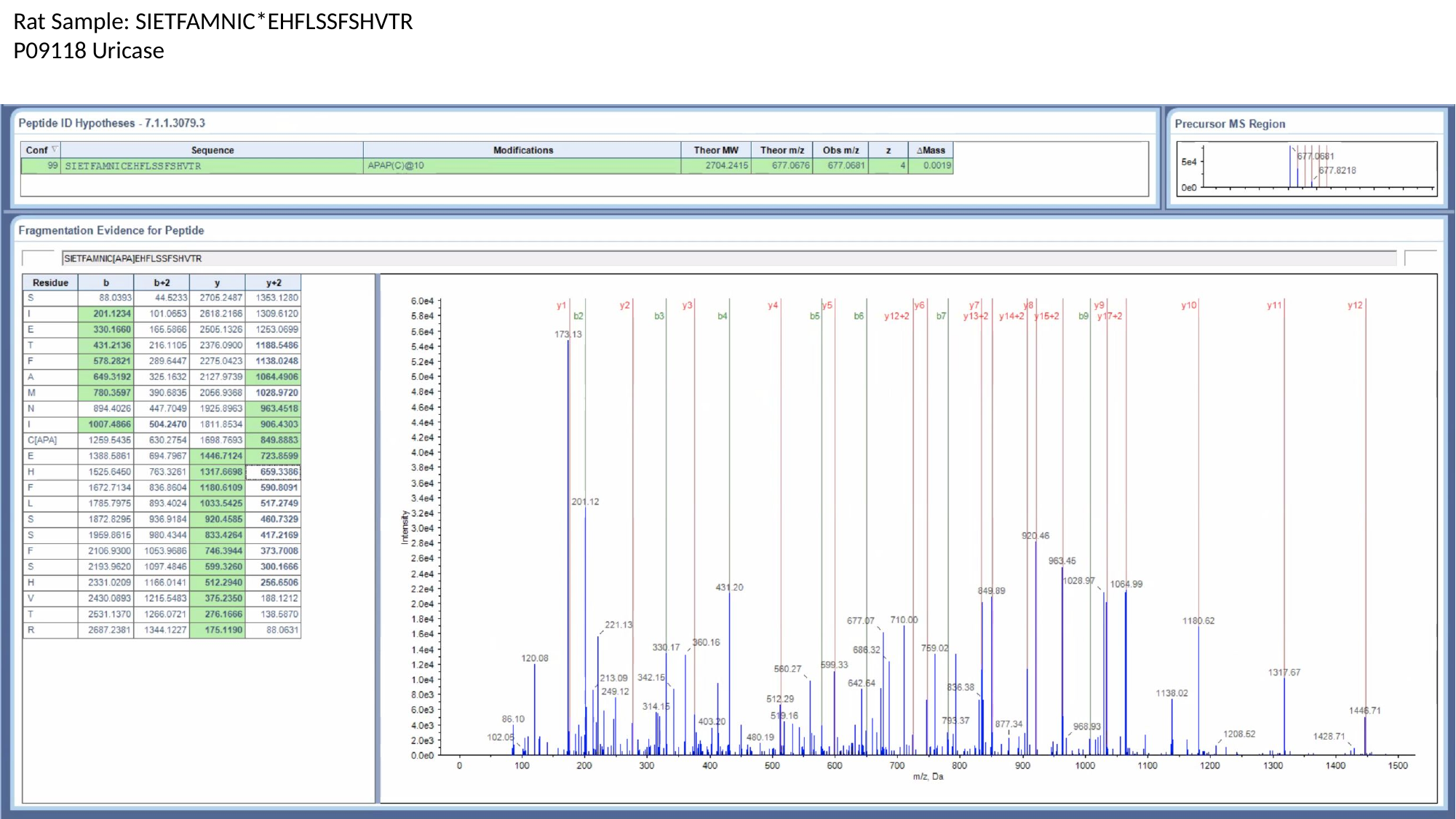

Rat Sample: SIETFAMNIC*EHFLSSFSHVTR
P09118 Uricase

## Slide 34
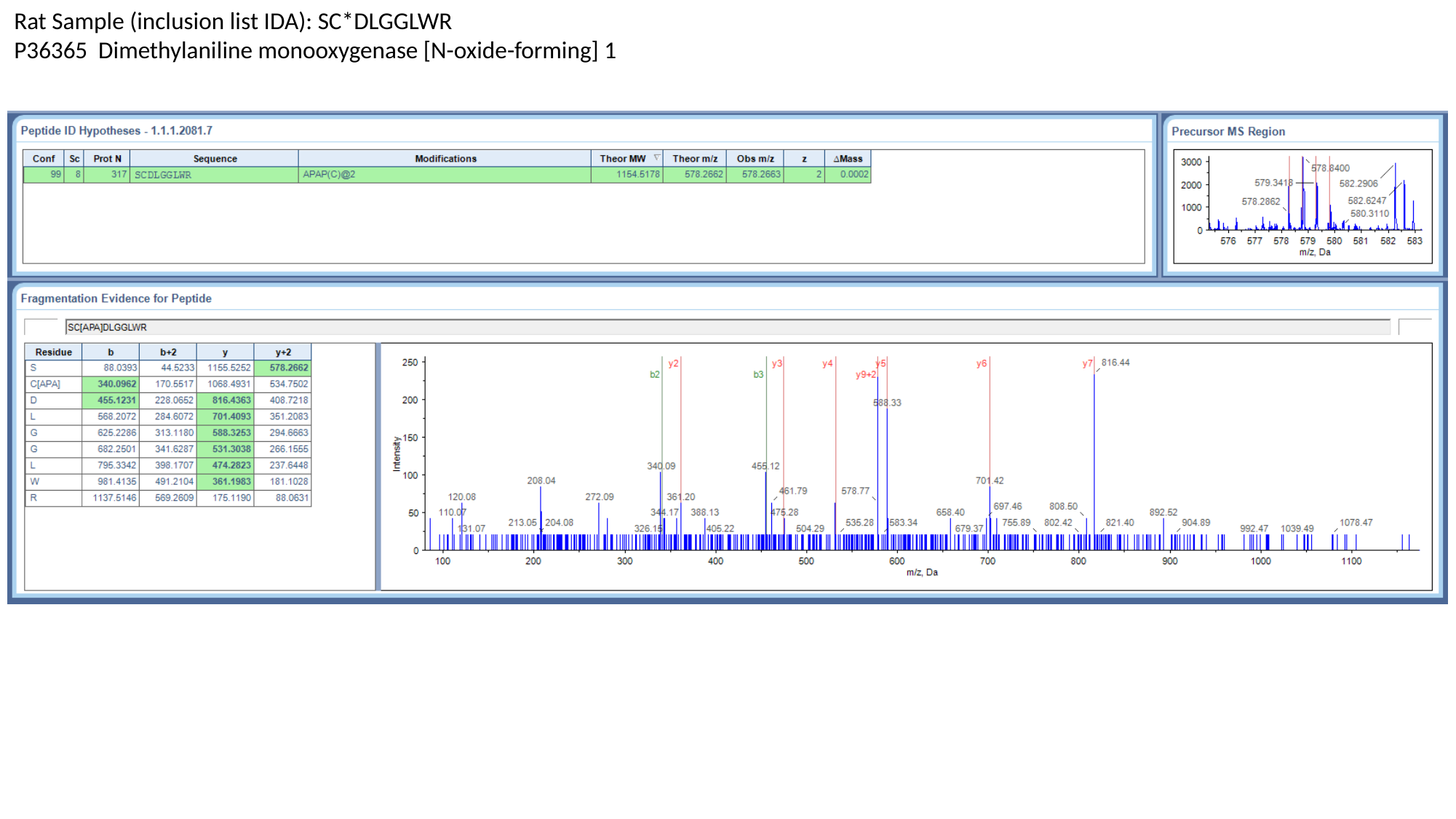

Rat Sample (inclusion list IDA): SC*DLGGLWR
P36365 Dimethylaniline monooxygenase [N-oxide-forming] 1
